# Genome mining reveals the unique function of UbiA-type prenyltransferase in *Laetiporus sulphureus*

**DOI:** 10.1007/s13659-025-00571-2

**Published:** 2026-01-11

**Authors:** Yue Wang, Qian Wang, Chunlei Wang, Pengchao Wang, Ran Wang, Jing Wu, Hirokazu Kawagishi, Chengwei Liu

**Affiliations:** 1 State Key Laboratory of Utilization of Woody Oil Resource, College of Life Science, Northeast Forestry University, Harbin, 150040 China; 2 College of Food and Biotechnology, Changchun Polytechnic University, Changchun, 130033 China; 3 Faculty of Agriculture, Iwate University, 3-18-8 Ueda, Morioka, 020-8550 Japan; 4 Faculty of Agriculture and Research Institute of Mushroom Science, Shizuoka University, Shizuoka, 422-8529 Japan

**Keywords:** *Laetiporus sulphureus*, UbiA prenyltransferase, Heterologous expression, Targeted mutation, *Aspergillus oryzae*

## Abstract

Eight UbiA-type prenyltransferases were mined in *Laetiporus sulphureus* by bioinformatic analysis, and phylogenetic analysis showed that they have unique functions. Through heterologous expression in *Aspergillus oryzae* and addition of exogenous hydroquinone (HYQ, **2**) substrate, it was found that *LaPT3* could transfer isoprenyl groups on **2**. Substrate specificity studies revealed that *LaPT3* was substrate specific and could only transfer dimethylallyl diphosphate (DMAPP) using **2** as substrate to produce the product 2-(3-methylbut-2-en-1-yl) benzene-1,4-diol (**1**). The key active sites of LaPT3 were analyzed and two key amino acid sites near the conserved motifs were targeted for mutation, and the product yields were reduced to 60% and 29% respectively by mutating N100 to S and G208 to A. Molecular docking and site-directed mutagenesis results indicate that these two amino acid sites play a crucial role in the catalytic generation of **2** by LaPT3 to produce **1**.

**Graphical Abstract:**

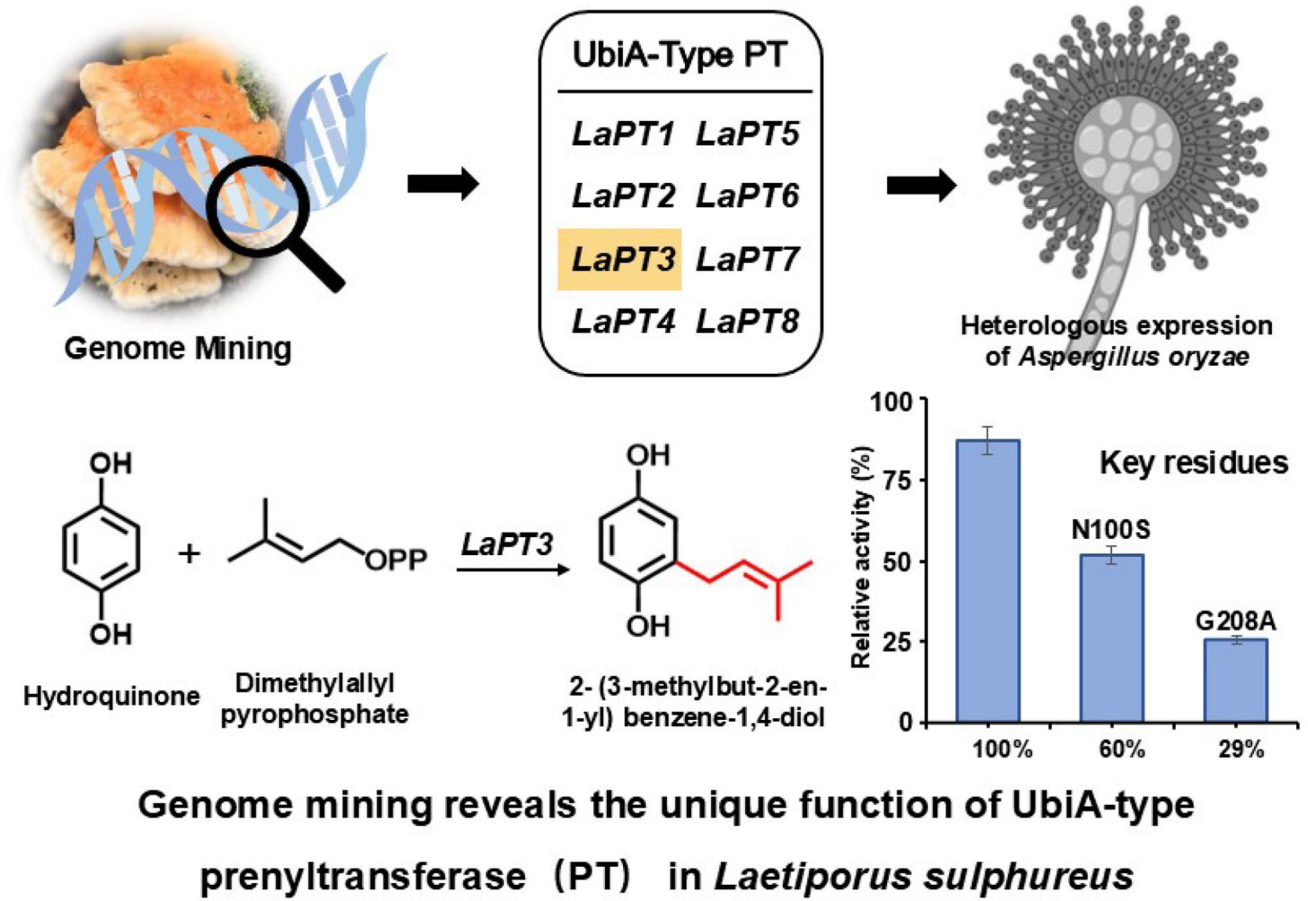

**Supplementary Information:**

The online version contains supplementary material available at 10.1007/s13659-025-00571-2.

## Introduction

*Laetiporus sulphureus* is a representative species of the genus *Laetiporus*, which belongs to the family Polyporaceae, order Polyocracies, order Agaricomycetes, and is widely distributed in the decayed parts of deciduous tree species in Europe, South America, Africa, and Asia, and is a wood-decay fungus. *L. sulphureus* is known as the “chicken in the forest” because of its fruiting bodies with bright and attractive colors, fresh and tender meat, and rich fatty acids, lipids and bioactive metabolites [8]. It has been used as food and herbal medicine for a long time in Asia. It is a medicinal and edible macrofungus with important edible value [17]. Fungi of *Laetiporus* also produce a variety of bioactive metabolites, including polysaccharides, melanin, lectins, sterols, terpenes, saponins, phenolic acids and tocopherols [30–32]. These metabolites have significant medicinal values such as anti-microbial, anti-tumor, anti-inflammatory, anti-clotting, anti-oxidant, antibacterial and cytostatic activities [14, 15, 18, 28]. The in-depth study of *L. sulphureus* and its metabolites provides a broad prospect for its application in the fields of medicine, food and biotechnology, and demonstrates its great potential as a raw material for natural medicine and functional foods [6].

Prenyltransferases (PT) are a class of enzymes that catalyze isoprenylation reactions requiring the involvement of isoprenyl donors and acceptors. Common donors are isoprenyl pyrophosphate (XPP, X represents isoprenyl chains of different lengths), including GPP, FPP, GGPP, etc. [13]. Based on their evolutionary origin, structure and intracellular localization, PTs can be classified into two groups: soluble prenyltransferases (sPTs) and membrane-bound PTs (UbiA) [33]. UbiA is a membrane-bound prenyltransferase that depends on biological membranes for its function, catalyzes the production of a number of secondary metabolites that are not essential for the growth and development of organisms, and isoprenylates many essential bioactive compounds that must be isoprenylated in order for them to be solubilized in biological membranes and perform their functions [12, 27]. UbiA prenyltransferase (UbiA-PTs) comprises at least one aspartic acid-rich motif (e.g., NQxxDxxxD) for binding isoprenyl pyrophosphate, and its catalytic activity is typically dependent on the assistance of metal ions to maintain the stability of the enzyme’s structure and to facilitate the isoprenylation reaction [19, 25].

In recent years, some UbiA-PTs have been successively identified in fungi, which are able to use aromatic compounds as substrates for isopentenyl transfer at different positions [38]. However, not many UbiA-PTs have been identified in the basidiomycetes, only nine have been found, and the vast majority of these enzymes are capable of transferring isopentenyl groups using only 4-hydroxybenzoic acid (PHB, **3**) as a substrate, and only a few of them are capable of using other aromatic compounds as a substrate. For example, UbiA-type PTs (VibP1 and VibP2) from *Boreostereum vibrans* can accept **3** as an isopentenyl acceptor for isoprenylation with GPP and FPP as isopentenyl donors. In addition, VibP1 and VibP2 can undergo isoprenylation with ShPT1 and ShPT2 from *Stereum hirsutum* using **3**, p-hydroxybenzyl alcohol (**4**) and *p*-hydroxybenzaldehyde as substrates [7]. UbiA-type PT (PanE) from *Panus rudis* is capable of isoprenylation using **3** as substrate and DMAPP as donor [37]. The discovery of ClaS is considered a groundbreaking turning point, as researchers have found that UbiA-type PT (ClaS) from *Clitocybe clavipes* is an atypical PHB-type UbiA prenyltransferase, which is able to isoprenylation with **2** rather than **3** as the acceptor, and GPP as the donor [34]. Recently, our team has elucidated the key prenyltransferase step in the biosynthetic pathway of hericenones from *Hericium erinaceus*. *Aspergillus oryzae* has emerged as a crucial tool in the study of fungal natural product biosynthesis [21]. Through heterologous expression in *A. oryzae*, it was first demonstrated that the UbiA-type PT enzyme HePT8 catalyzes the GPP transfer in orsellinic acid, synthesizing the core skeleton intermediate for hericenone type compounds [9].

In the present study, we found that LaPT3 from *L. sulphureus*, is also an atypical PHB-type UbiA-PT, which is able to undergo isoprenylation with **2**, but not **3**, as the acceptor and DMAPP as the donor to produce 2- (3-methylbut-2-en-1-yl) benzene-1,4-diol (**1**). Targeted mutagenesis of two key amino acid sites near the conserved motif of LaPT3 revealed that the product yields were reduced to 60% and 29%, respectively, LaPT3 is the first UbiA-PT reported in fungi that uses **2** as an acceptor to transfer DMAPP. The discovery of the key active site provides a theoretical basis for the subsequent rational design of modified UbiA-PT. Our study further enriches the library of natural producers of **1**, which may provide valuable genetic elements and strain resources for the development of engineered strains of these compounds.

## Results

### Identification and analysis of *L. sulphureus* UbiA-PT sequences

**Fig. 1:**
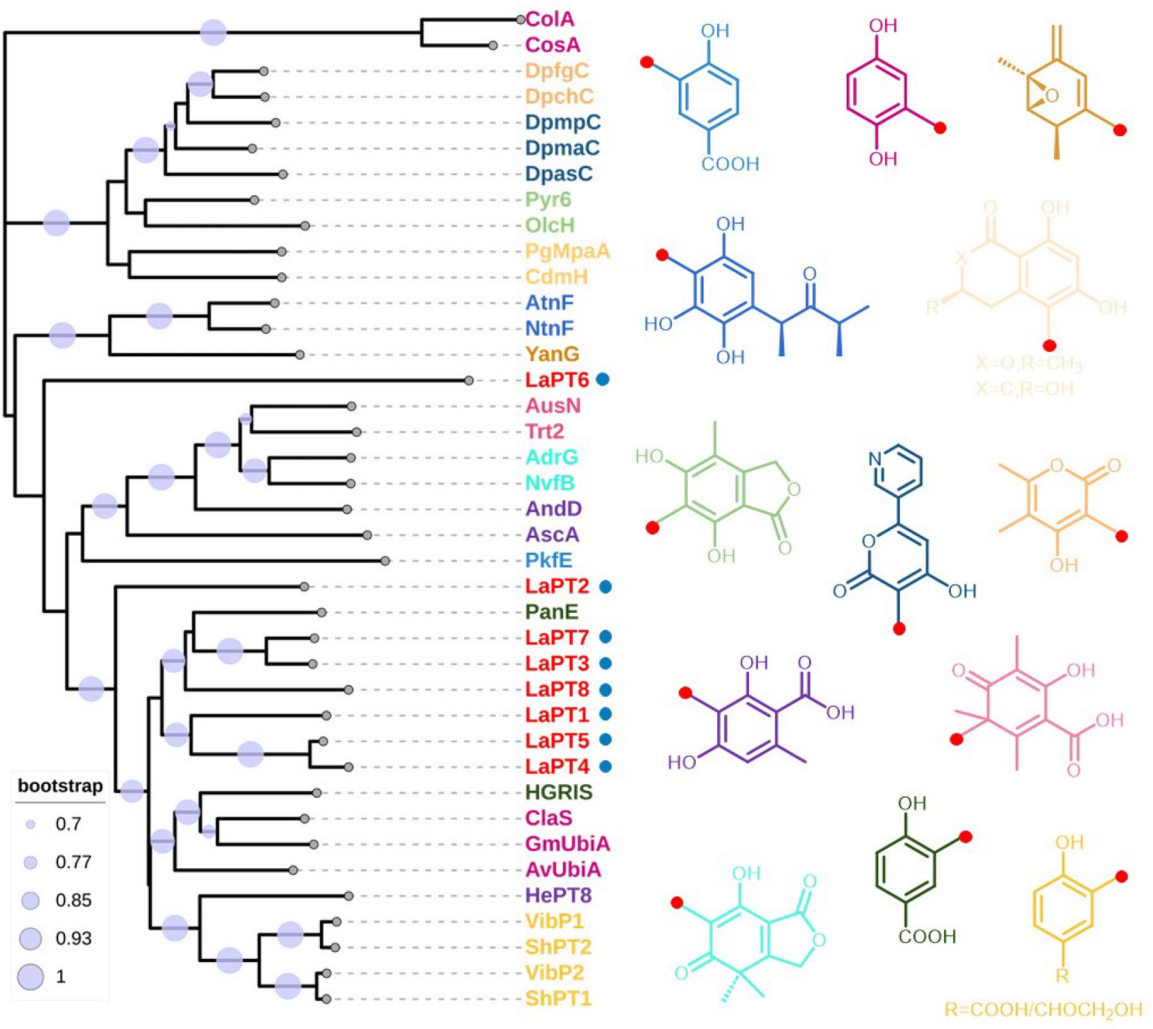
Phylogenetic analysis and substrate structure of fungal UbiA prenyltransferase (*red dots* represent the position of isoprenylation)

Using the already characterized UbiA-PT *ClaS* as a target gene [34], the *L. sulphureus* genome was blasted, and eight genes potentially encoding UbiA-PT were screened, named *LaPT(1–8)*. Analysis of the gene structure of *LaPTs* revealed that these genes ranged in size from 1000 to 1300 bp, with the number of introns ranging from 0 to 7 (Fig. S1), and the length of the encoded proteins was in the range of 263–362 amino acids (Table S2). The transmembrane segments of LaPTs were analyzed, and it was found that those LaPTs with 5–8 transmembrane structural domains belong to insoluble prenyltransferase, which are classified as UbiA class PTs (Fig. S2). To further understand the function of the functional family of LaPTs in *L. sulphureus*, the conserved structural domains of eight proteins in *L. sulphureus* were analyzed, and it was found that except for LaPT6 the other seven UbiA-PTs have the three conserved motifs (NDxxDxxxD, YxxxK and DxxxD) of the UbiA transferase [24]. It indicates that these seven LaPTs may have the ability to catalyze isoprenyl transfer, while LaPT6 is unable to perform isoprenyl transfer (Fig. S3). In order to better analyze the evolutionary properties of *L. sulphureus* UbiA-PT, LaPTs were phylogenetically analyzed with UbiA-PT genes from functionally known fungi. Phylogenetic analysis showed that proteins with the same function were clustered in the same branch, with LaPT2 and LaPT6 each belonging to a separate branch, and LaPT1, LaPT3, LaPT4, LaPT5, LaPT7, and LaPT8 in the same branch, predicted to possibly have the same function (Fig. 1, Table S3). The relative phylogenetic independence of these genes from UbiA-PT, whose function is known, suggests that they may have different donors and may catalyze different substrates to generate products with more diverse structures.

### Functional analysis of *LaPT(1–8)* in *A. oryzae*

**Fig. 2:**
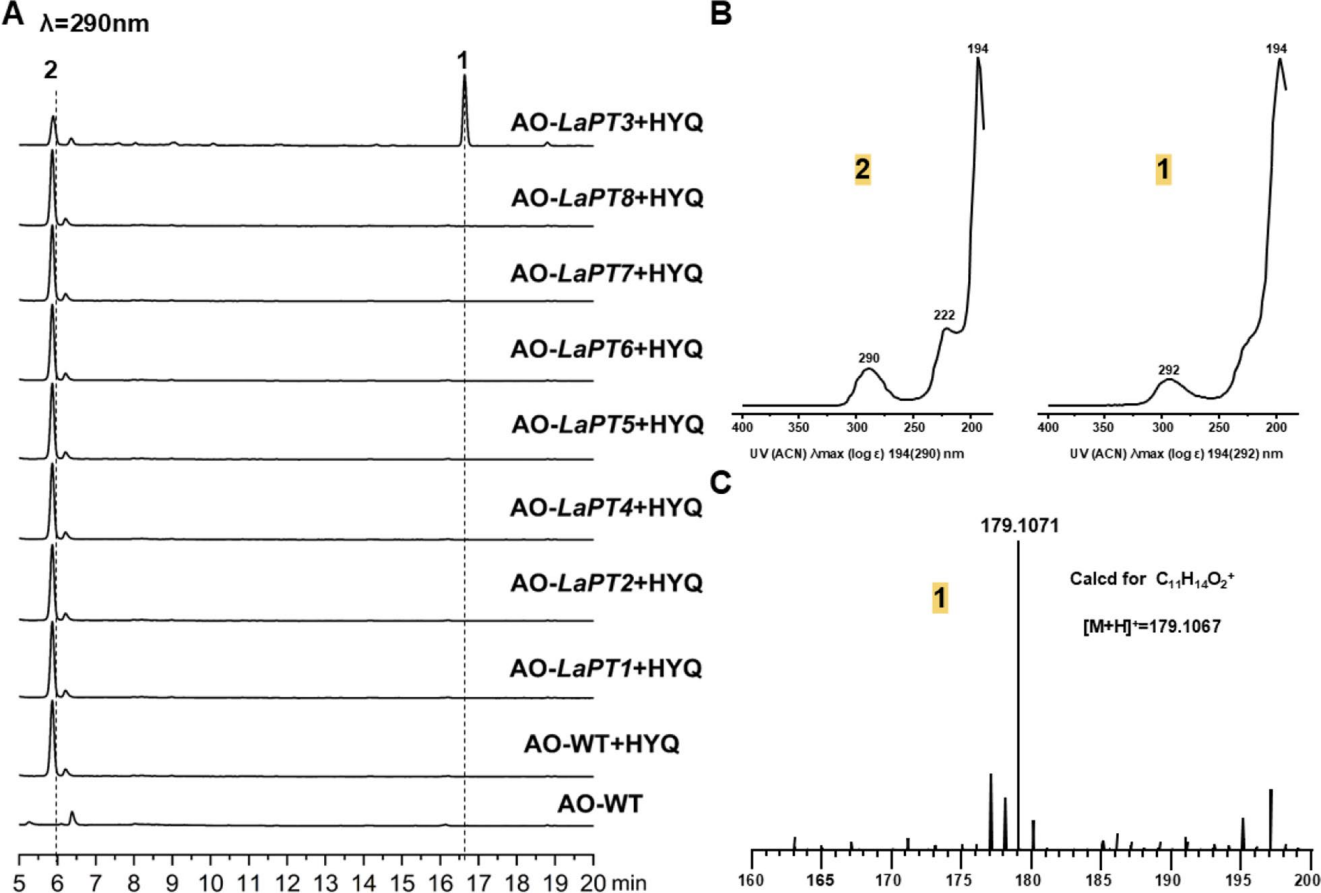
HPLC and LC–MS profiles. **A** HPLC detection of AO-*LaPT (1–8)* fed **2**; **B** UV spectra of **2** and **1**; **C** mass spectra of compound **1**

To associate metabolites with the eight LaPTs genes encoding proteins, we amplified these genes from sulfur-oxidizing fungal genomic DNA. Purified PCR fragments were cloned into the pDP201 vector to construct the pDP201-*LaPT (1–8)* plasmid. Given *A. oryzaes* ability to autonomously splice introns, the plasmid was transformed into the *A oryzae* strain NSPlD1 to obtain the AO*-LaPT (1–8)* transformant. In order to verify the functionality of *LaPT (1–8)*, we performed substrate feeding experiments using **2** and all the resulting transformants were cultured separately with 2 ml of MPY liquid mini-tube medium, as well as culturing wild-type *A. oryzae* as a control. The HPLC results showed that, compared to the wild-type (AO-WT), after feeding the substrate **2**, AO-*LaPT3* produced one new metabolite, while the amount of substrate **2** was significantly reduced compared to the control (Fig. 2a). UV mapping analysis showed that the UV absorption feature of compound **1** at 294 nm was similar to that of **2**, revealing that it might be a derivative of **2** (Fig. 2b). No new metabolites were detected to be generated in the fermentation products of AO-*LaPT (1–2, 4–8)*. Based on the own function of UbiA-PT, it was speculated that the new product might be an isopentenyl derivative of **2**, and in order to further verify our conjecture, the product was further analyzed and detected by LC–MS. The ionic peak of compound **1** was m/z 179.1071 m/z [M + H] ⁺, and the molecular weight exceeded that of **2** by 68 Da, suggesting that its structure contained an additional DMAPP unit with the molecular formula calcd. for C_11_H_14_O_2_ [M + H] ^+^: 179.1067. (Fig. 2c).

### Separation, extraction and structural characterization of compound 1

In order to confirm the structure of compound **1**, we performed a 2 L liquid fermentation of AO-*LaPT3*, and after 1 day of incubation at 30 °C, the substrate **2** (50 μg/mL, methanol solution) was added to the culture medium. After incubation at 30 °C for another 3 days, the bacterial liquid was extracted using ethyl acetate, and later isolated and purified using silica gel and HPLC to obtain compound **1**. It was detected using NMR after dissolving in deuterated chloroform (Figures S4–S8).^13^C-NMR (CDCl_3_, 125 MHz) *δ*: 128.23, 148.13, 116.45, 113.72, 149.31, 116.60) and ^1^H-NMR (CDCl_3_, 500 MHz) *δ*: 6.57 (1H, dd, *J* = 8.5, 3.0 Hz), 6.67 (1H, d *J* = 8.4Hz), 6.61 (1H, d* J* = 3.0Hz) data showed that compound **1** contains **2** unit and ^13^C-NMR showed 5 more C signals at positions C1 (*δ*: 29.70), C2 (*δ*: 121.42), C3 (*δ*: 134.96), C4 (*δ*: 25.80), and C5 (*δ*: 17.88) respectively for compound **1** on **2** basis. ^1^H-NMR showed two more methyl groups at positions C4 (1.77, 3H, s) and C5 (1.76, 3H, s), one methylene group (3.29, 2H, d *J* = 7.3Hz) and one hydrogen on the allyl group (5.29, 1H, m), respectively (Table 1). Consistent with isopentenyl signals, and this result is consistent with reported NMR patterns of the compound. Thus, we identified compound **1** as 2-(3-methylbut-2-en-1-yl) benzene-1,4-diol [36].

**Table 1:** The NMR spectral data of **1** (CDCl_3_)

Position	*δ*_H_ (*J* in Hz)	*δ*_C_
1′		128.23
2′		148.13
3′	6.57 (1H, dd *J* = 8.5, 3.0 Hz)	116.45
4′	6.68 (1H, d* J* = 8.5 Hz)	113.72
5′		149.31
6′	6.61 (1H, d* J* = 3.0 Hz)	116.60
1	3.29 (2H, d* J* = 7.0 Hz)	29.70
2	5.29 (1H, m)	121.42
3		134.96
4	1.77 (3H, s)	25.80
5	1.76 (3H, s)	17.88

### Substrate specificity studies of LaPTs

**Fig. 3:**
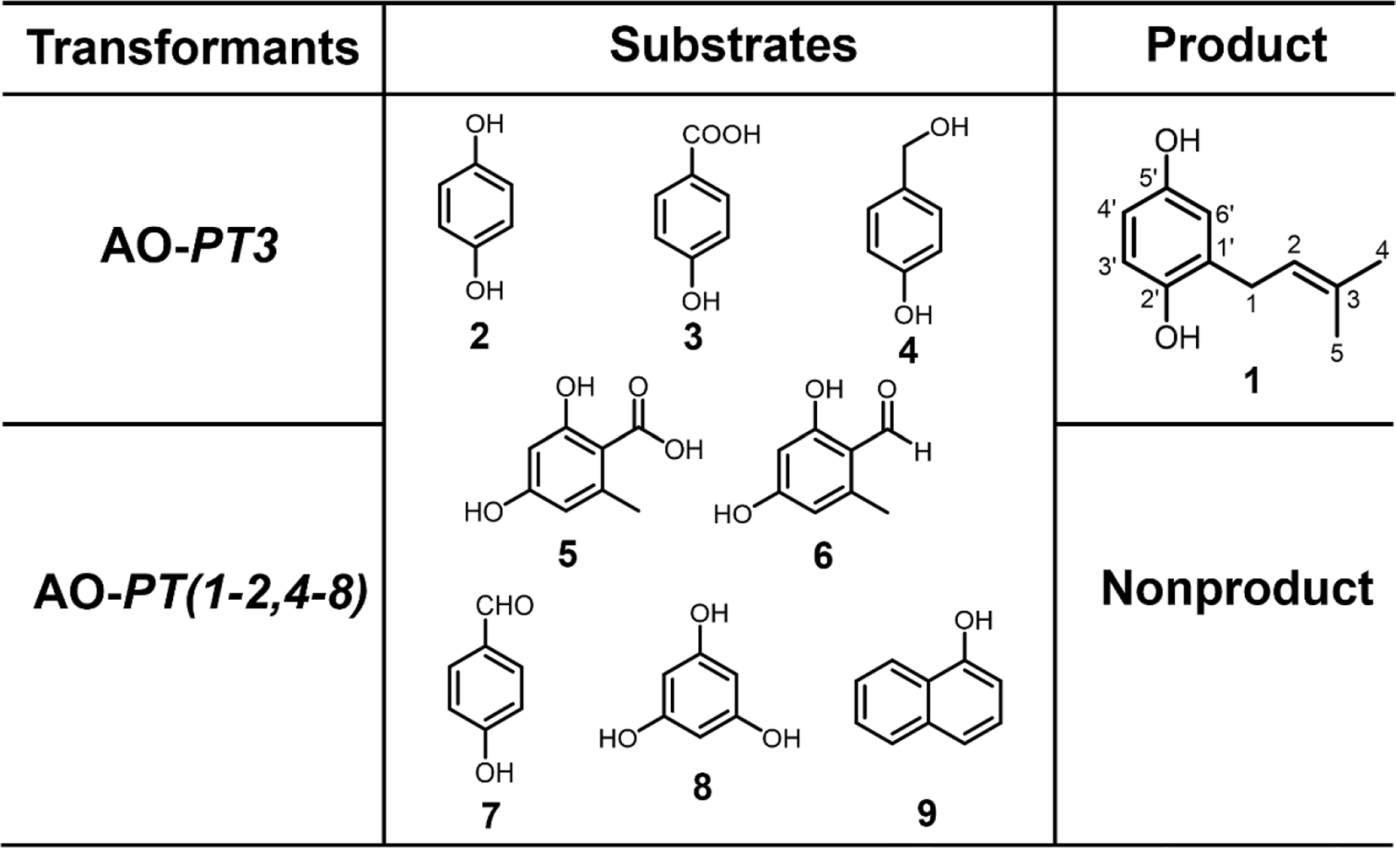
Substrate specificity analysis of AO-*LaPT (1–8)*

Most fungal PTs are substrate selective, which is the basis for the structural and functional diversity of natural products. To investigate the substrate specificity of *LaPT (1–8)*, eight structurally similar compounds were selected as potential substrates and added to the culture system of AO-*LaPT (1–8)* strains for experiments (Fig. 3). HPLC assay revealed that only when **2** was used as the substrate, AO-*LaPT3* catalyzed the isoprenylation of **2** to produce compound **1**, when 2 serves as the substrate; no products were detected for the remaining seven tested substrates. This suggests that *LaPT3* exhibits substrate specificity for **2**, and the enzyme can only catalyze the isoprenylation reaction with **2** as the receptor, a property that is consistent with the reported substrate-selective pattern of fungal UbiA-PT.

### LaPT3 key active site analysis

**Fig. 4:**
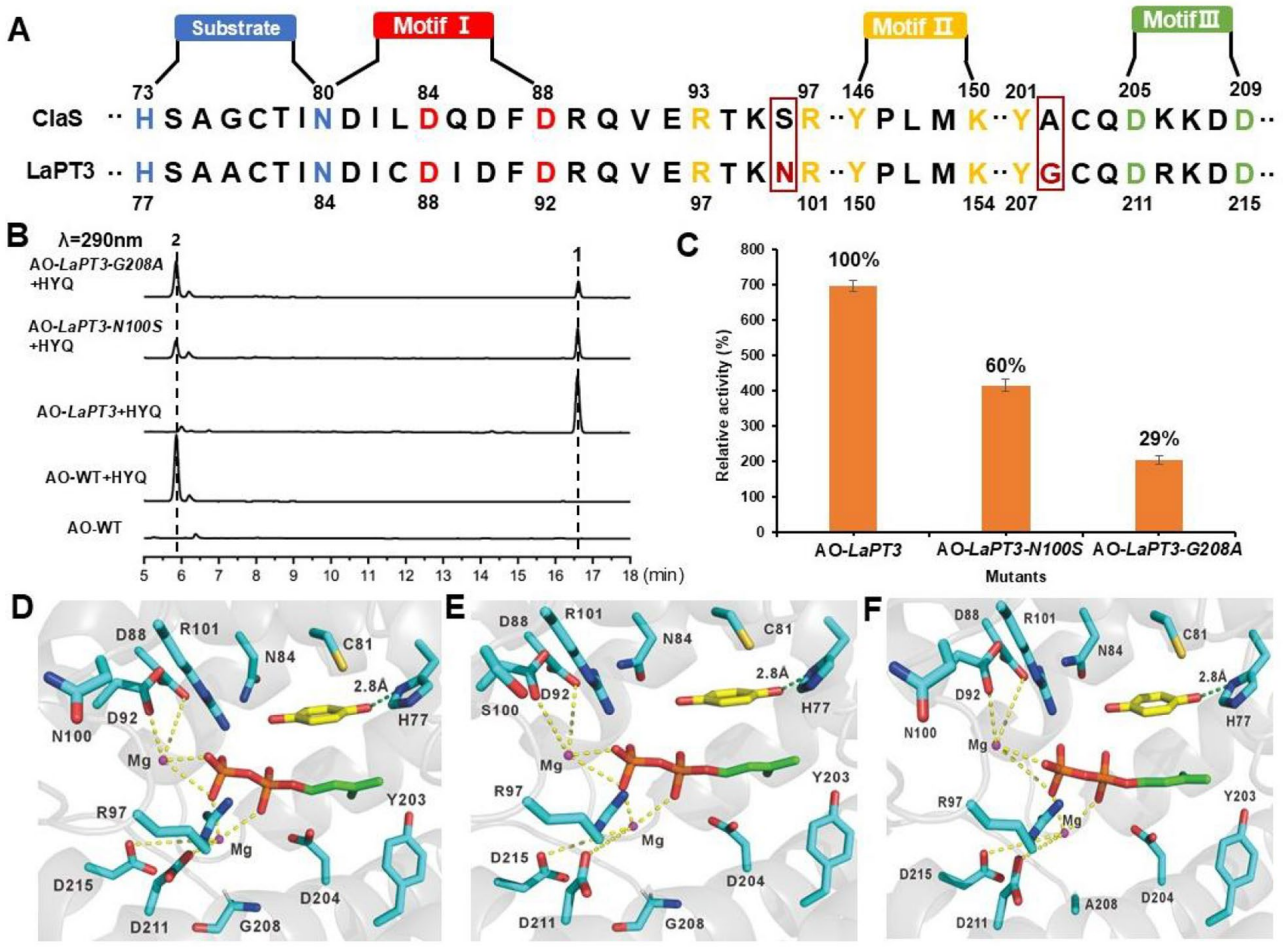
Key amino acid site mutations of LaPT3, HPLC profiles and yield analysis. **A** Schematic diagram of ClaS and LaPT3 conserved motifs and key amino acid residues analyzed and mutated; **B** HPLC profile of the AO-*LaPT3* mutant at residues 100 and 208 fed with **2**; **C** The relative enzyme activities for AO-*LaPT3* mutant strains; **D** Schematic diagram of LaPT3 docking results with Mg^2^⁺ ions and DMAPP molecules; **E** Schematic diagram of the docking results between LaPT3 (N100S) and Mg^2^⁺ ions and DMAPP molecules; **F** Schematic diagram of the docking results between LaPT3 (G208A) and Mg^2^⁺ ions and DMAPP molecules (*yellow* represents Substrate **2**, while *red* and *green* represent DMAPP)

UbiA-PT possesses three typical conserved motifs, NDxxDxxxD, DxxxD, and YxxxK, for Mg^2+^ ion and isoprenyl group binding. Multiple sequence alignment reveals that LaPT3 and ClaS share the same conserved motifs but perform distinct functions. As an atypical PHB-type UbiA PT, ClaS transfers GPP to position 2, whereas LaPT3 transfers DMAPP to position 2. We hypothesize that neighboring amino acids within these conserved motifs confer the ability to transfer different substrates to these two enzymes. We identified amino acid differences between LaPT3 residues 100 and 208 and ClaS, with this region residing within the substrate-binding domain. Therefore, we mutated N to S at position 100 and G to A at position 208 to confer LaPT3 with the ability to transfer other isoprenyl groups (Fig. 4a).

The constructed mutant recombinant plasmid was introduced into *A. oryzae* to obtain the AO-*LaPT3-N100S* and AO-*LaPT3-G208A* mutant transformants. The mutant transformants were fed **2** separately to detect the changes in isoprenylation function (Fig. 4b). Unfortunately, that neither position 100 nor position 208 of the mutants was not able to transfer other isoprenyl group. However, that the isoprenylation activity of the mutant underwent a significant reduction. Mutating N100 to S reduced the product yield to 60% of the original yield, and mutating G208 to A reduced the product yield to 29% of the original product yield (Fig. 4c). Based on the analysis of the ApUbiA protein structure (PDB ID: 4OD5) [2], we modeled DMAPP and HYQ in the LaPT3 structure using AutoDock software. Two magnesium ions (Mg^2^⁺) coordinate through four aspartic acid residues (D88, D92, D211, and D215) within two conserved aspartic acid-rich motifs (Fig. 4d), which collectively stabilize the diphosphate moiety of DMAPP. The N100 residue was found to be in close proximity to D88 and D92. Mutating it to S further disrupted the binding of D92 to Mg^2^⁺, resulting in reduced yield of the N100 S mutant (Fig. 4e). The G208A mutant exhibited an even greater reduction in yield, indicating that larger residues hinder substrate binding or protein folding (Fig. 4f). These results demonstrate that amino acids adjacent to the conserved motif also play a crucial role in maintaining enzyme activity.

## Conclusion and discussion

As a woody macrofungus with high medicinal and edible value, the fruiting bodies of *L. sulphureus* have a long history of consumption in China, Europe and the Americas, and their medicinal value has been documented in ancient Chinese medical texts [29]. In recent years, many structurally diverse and biologically active natural products, such as sesquiterpenoids, triterpenoids, sterols and polyenes, have been isolated from *L. sulphureus* [3, 4, 10, 11, 16, 20], they have a variety of physiological activities such as antitumor, antifungal, anticancer and anti-inflammatory. And because of the known cultivation methods of this mushroom, together with its rich chemical composition and beneficial effects on the human body, many companies consider it as a potential raw material for the food industry [1].

UbiA-PTs is a membrane-bound prenyltransferase that is dependent on biological membranes and is able to participate in cellular processes and multiple metabolic pathways, resulting in structurally and biologically diverse natural products, and is therefore expected to be a potential target for new drug discovery [2, 19, 35]. In plants UbiA-PTs produces many natural products with enhanced pharmacological activity, such as cannabinoids, polycyclic polyprenylated acylphloroglucinols, isoprenylated flavonoids and stilbenoids [22]. In macrofungus in identified a number of UbiA-PTs, most of which are capable of using aromatic compounds as substrates and transferring different amounts of isoprenyl groups at different positions to produce a number of meroterpenoids precursor substances. Subsequently, more structurally complex meroterpenoids are generated by oxidoreductases. The introduction of isoprenyl groups increases the structural diversity and biological activity of these natural products. We therefore used a genome mining strategy to target UbiA-PTs in *L. sulphureus*, and a total of eight UbiA-PTs were mined, and phylogenetic analyses suggested that they may have unique functions. LaPT3 was found to transfer DMAPP on **2** by heterologous expression in *A. oryzae* and exogenous substrate addition. The substrate specificity of LaPT3 reveals its potential application value in natural product synthesis and drug development, providing valuable ideas for the design and development of enzymes with specific substrates. By analyzing the key active site of LaPT3, as well as performing targeted mutagenesis of its key amino acid sites, mutating N100 to S and G208 to A of LaPT3, we found that there was a significant decrease in the yield of the products in both cases, suggesting that we have found the key active site for controlling LaPT3. The discovery of key active sites provides a molecular foundation and theoretical basis for the subsequent rational design of modified PTs.

These findings provide an important foundation for the development of novel isoprenylated biocatalysts. In the future, we can continue to excavate heteroterpene post-biosynthesis modification genes and targeted modification of related enzyme genes in the *L. sulphureus* genome to optimize the efficiency of meroterpene synthesis and enhance the substrate specificity and catalytic activity. By combining synthetic biology and metabolic engineering technologies, we can realize the efficient biosynthesis of these valuable compounds.

## Materials and methods

### Strain and medium

Standard recombinant DNA techniques were used for molecular cloning using *Escherichia coli* DH5α. The fungal strain used in this experiment was *L. sulphureus* NWAFU-1 [5]*.* The heterologous expression host strain used in this study was *A. oryzae* NSPlD1 in Czapek-Dox (CD) + Dextrin [0.3% NaNO_3_, 0.2% KCl, 0.1% KH_2_PO_4_, 0.05% MgSO_4_‧7H_2_O, 0.002% FeSO_4_‧7H_2_O, 2% Dextrin, 0.9% (NH_4_)_2_SO_4_, pH 6.0, 100 mL] with necessary supplements (0.15% methionine, 0.5% uridine, 0.2% uracil) in the medium for cultivation of nutritive strains of *A. oryzae* [26]. The transformants were grown on DPY (dextrin-polypeptone-yeast extract: 2% dextrin, 1% polypeptone, 0.5% yeast extract, 100 mL) medium supplemented with appropriate nutrients [23].

### Extraction of gDNA and cDNA

The 5 × 5 mm *L. sulphureus* clusters were inoculated in 100 mL of Potato Dextrose Broth and incubated for 5–7 days at 28 °C and 200 rpm with oscillation to collect the mycelium, and then the genome of *L. sulphureus* was extracted using chloroform extraction. Total RNA was extracted from dried mycelium using TRIzol reagent (Invitrogen) according to the manufacturer’s instructions, followed by reverse transcription with DNase I (Life Technologies). The cDNA was synthesized with the PrimeScript II First Strand cDNA Synthesis Kit (TaKaRa) using oligonucleotide (dT) primers according to the manufacturer’s instructions.

### Bioinformatics analysis

Based on *L. sulphureus* genome-wide data [5], a blast search was performed in the *L. sulphureus* genome with Bioedit using the already characterized UbiA-PTs *ClaS* as a target gene, and a total of eight UbiA-PTs were identified, and the web analysis tool 2ndFind (http://biosyn.nih.go.jp/2ndFind/) identified eight genes for UbiA-PTs from *L. sulphureus*, named *LaPT (1–8)*. Multiple sequence comparison was performed using MEGA11, and ESPript3.0 (https://espript.ibcp.fr/ESPript/ESPript/index.php) was used to visualize the results. Using MEGA11 software, neighbor-joining method was used to generate an evolutionary tree to determine its branches, and self-expansion values were calculated based on 1000 replicates. The transmembrane structural domains of *LaPT (1–8)* were analyzed by TMHMM online analysis website.

### Plasmid construction and transformation of *A. oryzae*

*LaPT (1–8)* was amplified from *L. sulphureus* genomic DNA using the primer sets shown in Supplemental Table S1. PCR reactions were performed using KOD-Plus-Neo (TOYOBO). Then introduced into the pDP201 vector using a ClonExpress Ultra One Step Cloning Kit (Vazyme Biotech Co., Ltd.) to construct expression plasmids, pDP201-*LaPT (1–8)*. A spore suspension of *A. oryzae* NSPLD1 (1.0 × 10^8^ cells) was inoculated into CD (0.3% of NaNO_3_, 0.2% of KCl, 0.1% of K_2_HPO_4_, 0.05% of MgSO_4_‧7H_2_O, 2% of dextrin, 0.002% of FeSO_4_‧7H_2_O, 0.15% of methionine, 0.9% of (NH_4_)_2_SO_4_, 0.49% of uracil, 0.2% of uridine, 100 mL, pH 5.5) in medium and incubated at 30 °C, 200 rpm for 2 days. Mycelium were collected, and the mycelium was washed 3–5 times with distilled water to remove the water. Protoplasts were incubated for 2 h at 30 °C using Yatalase (Takara; 5.0 mg/mL) in solution 1 (0.8 mM NaCl, 10 mM NaH_2_PO_4_, pH 6.0). The protoplasts were centrifuged at 800 g for 5 min and washed with 0.8 M NaCl solution and repeated three times. The protoplasts were then conditioned to 2.0 × 10^8^ cells/mL by adding solution 2 (0.8 M NaCl, 10 mM CaCl_2_, 10 mM Tris–HCl, pH 8.0) and solution 3 (40% (w/v) PEG 4000, 50 mM CaCl_2_, 50 mM Tris–HCl, pH 8.0) at a volume ratio of 4/1. The pDP201-cas9 plasmid (2 μg) and pDP201-*LaPT (1–8)* plasmid (2 μg) were added to the protoplast solution (200 μL). Aliquots were incubated on ice for 20 min, and then solution 3 (1 mL) was added to the aliquots. After incubation for 20 min at room temperature, Solution 2 (10 mL) was added to the mixture and the mixture was centrifuged at 800 g for 10 min to remove the supernatant. After decantation, the residue was diluted with Solution 2 (1 mL) and the mixture (200 μL) was poured onto a CD agar plate (1.5%), which was then covered with soft-top CD agar (0.6%) containing 21.8% sorbitol. The plates were incubated at 30 °C for 3–5 days.

### Biotransformation experiments

Mycelium of the transformant strain AO-*LaPT (1–8)* was inoculated into 2 mL of MPY (maltose-peptone-yeast extract: 3% maltose, 1% polypeptone, 0.5% yeast extract) medium containing the appropriate nutrients, and incubated at 30 °C for 1 day. The substrate (**2**, **3**, **4**, olivanic acid **5**, o-orsellinaldehyde **6**, 4-hydroxybenzaldehyde **7**, phloroglucinol **8** and 1-hydroxynaphthalen **9**) in methanol solution (50 μg/mL) was then added to the medium. After incubation at 30 °C for another 3 days, the extract products were separated by adding ethyl acetate and soaking overnight, the crude extract was analyzed.

### Compound detection methods

An Agilent HPLC 1260 Infinity II liquid chromatograph was used in this experiment with a stationary phase column of Agilent 5 TC-C18 (2) (250 × 4.6 mm), The system was operated on a mobile phase of methanol and water (0.1% formic acid) with a linear gradient of 10–40% MeOH for 5 min, followed by a linear gradient of 40–80% MeOH for 15 min, 80–100% MeOH for 5 min, 100% MeOH for 5 min, and 100–10% MeOH for 8 min. The detection wavelength was 290 nm, and the flow rate was 1 ml/min for 38 min per sample.

LC–MS was analyzed using a TripleTOF 6600 mass spectrometer (AB/SCIEX, Milford, MA) and an HPLC system (AB/SCIEX). The scanning mode was set as continuous full scan with positive ions, and the scanning range was *m/z* = 120–1200. The liquid-phase C18 reversed-phase chromatographic column was a Kinetex C18 column (150 × 4.6 mm, 2.6 μm) with the column temperature controlled at 30 ℃, the detection wavelength was in the region of 200–600 nm, and the sample volume was 1 μL with a flow rate of 0.5 mL/min. The mobile phase A was ddH_2_O containing 0.01% formic acid, and the mobile phase B was acetonitrile containing 0.01% formic acid. The gradient elution program was set up as follows: in 0–5 min, the mobile phase B was increased from 10 to 100%, and in 5–10 min, 100% of the mobile phase B was kept unchanged.

### Large-scale biotransformation and product separation

Mycelium of the transformed strain AO-*LaPT3* was inoculated into 20 vials of 100 mL of MPY medium containing appropriate nutrients. After incubation at 30 °C for 1 day, the substrate **2** (50 μg/mL, methanol solution) was added to the medium. After incubation at 30 °C for another 3 days, the mycelium and bacterial liquid were separated by filtration. The mycelium was placed in a conical flask, soaked overnight with appropriate amount of ethyl acetate, and the concentrate was extracted three times with equal volume of ethyl acetate, and concentrated to finally obtain the mycelial extract; the mycelium was soaked with acetone and then filtered to remove the fermentation broth, and then the bacterium was collected and extracted three times in successive infusions, and then extracted three times with equal volume of ethyl acetate, and concentrated to finally obtain the mycelial extract.

The fermentation broth extract was separated using silica gel column chromatography. Hexane/ethyl acetate (10:1 → 1:1, v/v) gradient elution, the volume of eluent for each gradient was twice the column volume. The analytes were analyzed by using a combination of TLC and HPLC, and after combining the target fractions, the purification was carried out by applying a semi-preparative column, YMC-Triart C18 (specification: 250 mm × 10 mm, 5 μm). The target compounds obtained after repeated separation and purification were dissolved in Chloroform-d(Cambridge Isotope Laboratories, Inc) and then detected by NMR.^1^H- and ^13^C-NMR spectra were recorded on a Bruker AMX-500 spectrometer. Chemical shifts were reported as *δ* scale in ppm as an internal reference (CDCl_3_; ^1^H NMR = 7.26 ppm, ^13^C NMR = 77.16 ppm). Data are reported as follows: chemical shift, multiplicity (s = singlet, d = doublet, m = multiplet), coupling constant (Hz), and integration.

### Site-directed mutagenesis of LaPT3

The cDNA of AO-*LaPT3* strain was used as a PCR template, and two candidate amino acid residues were mutated by site-directed mutagenesis, i.e., N at position 100 mutated to S, G at position 208 mutated to A. Subsequently, the mutated sequences were constructed into the pDP201C vector, which resulted in the mutant recombinant plasmid. The constructed recombinant plasmid was introduced into *A. oryzae* to obtain the AO-*LaPT3-N100S* and AO-*LaPT3-G208A* mutant transformants.

### Molecular docking

The three-dimensional structure of the LaPT3 protein was predicted using AlphaFold3. The complete amino acid sequence of LaPT3 was submitted to the AlphaFold3 server for modeling. Among the generated models, the one with the highest predicted local distance difference test (pLDDT) score was selected as the initial structure for subsequent molecular docking studies. Molecular docking experiments were performed using Discovery Studio software. Based on the ApUbiA structure (PDB:4OD5), Discovery Studio was employed to dock two Mg^2+^ ions, DMAPP, and **2** into the predicted structure of LaPT3. Active sites were defined based on homology models and conserved residues. The final protein–ligand complexes were obtained by considering CDOCKER interaction scores alongside the orientations of DMAPP and **2**. Visualization of the final docking complexes was performed using PyMOL software.

## Supplementary Information

Additional file 1.

## Data Availability

All data generated or analyzed during this study are included in this published article and its supplementary information files.

## References

[1] Adamska I. The possibility of using sulphur shelf fungus (*Laetiporus sulphureus*) in the food industry and in medicine-a review. 2023. Foods. 10.3390/foods12071539. 10.3390/foods12071539 PMC10093887 37048360

[2] Chang HY, Cheng TH, Wang AH. Structure, catalysis, and inhibition mechanism of prenyltransferase. IUBMB Life. 2021;73(1):40–63. 33246356 10.1002/iub.2418 PMC7839719

[3] Davoli P, Mucci A, Schenetti L, Weber RW. *Laetiporic* acids, a family of non-carotenoid polyene pigments from fruit-bodies and liquid cultures of *Laetiporus sulphureus* (Polyporales, Fungi). Phytochemistry. 2005;66(7):817–23. 15797608 10.1016/j.phytochem.2005.01.023

[4] Devkota KP, Covell D, Ransom T, McMahon JB, Beutler JA. Growth inhibition of human colon carcinoma cells by sesquiterpenoids and tetralones of *Zygogynum calothyrsum*. J Nat Prod. 2013;76(4):710–4. 23517126 10.1021/np400042q PMC3719390

[5] Dong WG, Wang ZX, Feng XL, Zhang RQ, Shen DY, Du S, et al. Chromosome-level genome sequences, comparative genomic analyses, and secondary-metabolite biosynthesis evaluation of the medicinal edible mushroom *Laetiporus sulphureus*. Microbiol Spectr. 2022;10(5):e0243922. 36200896 10.1128/spectrum.02439-22 PMC9602373

[6] Duan Y, Qi J, Gao JM, Liu C. Bioactive components of *Laetiporus* species and their pharmacological effects. Appl Microbiol Biotechnol. 2022;106(18):5929–44. 36063176 10.1007/s00253-022-12149-w

[7] Feng KN, Zhang Y, Zhang M, Yang YL, Liu JK, Pan L, et al. A flavin-monooxygenase catalyzing oxepinone formation and the complete biosynthesis of vibralactone. Nat Commun. 2023;14(1):3436. 37301868 10.1038/s41467-023-39108-x PMC10257657

[8] Grienke U, Zöll M, Peintner U, Rollinger JM. European medicinal polypores--a modern view on traditional uses. J Ethnopharmacol. 2014;154(3):564–83. 24786572 10.1016/j.jep.2014.04.030

[9] Han H, Peng S, Yang Y, Lin C, Wang P, Li C, et al. Discovery and biochemical characterization of prenyltransferases in the biosynthetic pathway of hericenones from *Hericium erinaceus*. Bioorg Chem. 2025;164:108822. 40759078 10.1016/j.bioorg.2025.108822

[10] Hassan K, Matio Kemkuignou B, Stadler M. Two new triterpenes from basidiomata of the medicinal and edible mushroom, *Laetiporus sulphureus*. Molecules. 2021. 10.3390/molecules26237090. 34885672 10.3390/molecules26237090 PMC8658958

[11] He JB, Tao J, Miao XS, Feng YP, Bu W, Dong ZJ, et al. Two new illudin type sesquiterpenoids from cultures of *Phellinus tuberculosus* and *Laetiporus sulphureus*. J Asian Nat Prod Res. 2015;17(11):1054–8. 26000880 10.1080/10286020.2015.1040774

[12] Heide L. Prenyl transfer to aromatic substrates: genetics and enzymology. Curr Opin Chem Biol. 2009;13(2):171–9. 19299193 10.1016/j.cbpa.2009.02.020

[13] Huang Y, Liu J, Yang B. Catalytic mechanism and engineering of aromatic prenyltransferase: a review. Int J Biol Macromol. 2025;313:144214. 40379159 10.1016/j.ijbiomac.2025.144214

[14] Jen CI, Lu MK, Lai MN, Ng LT. Sulfated polysaccharides of *Laetiporus sulphureus* fruiting bodies exhibit anti-breast cancer activity through cell cycle arrest, apoptosis induction, and inhibiting cell migration. J Ethnopharmacol. 2024;321:117546. 38061441 10.1016/j.jep.2023.117546

[15] Jovanović MM, Virijević K, Grujić J, Živanović M, Šeklić DS. Extract of edible mushroom *Laetiporus sulphureus* affects the redox status and motility of colorectal and cervical cancer cell lines. Biol Life Sci Forum. 2021;6(1):82.

[16] Khalilov Q, Li L, Liu Y, Liu W, Numonov S, Aisa HA, et al. Brassinosteroid analogues from the fruiting bodies of *Laetiporus sulphureus* and their anti-inflammatory activity. Steroids. 2019;151:108468. 31400389 10.1016/j.steroids.2019.108468

[17] Korabel IM, Panchak LV, Zyn AR, Vrubel OR, Antonyuk VO. Study of lipophilic substances of *Laetiporus sulphureus* (Bull. Fr) Murril at different stages of maturity of mushroom fruiting bodies. Biomed Chromatogr. 2025;39(7):e70140. 40515406 10.1002/bmc.70140

[18] Kosanić M, Petrovic N, Šeklić D, Živanović M, Kokanović M. Bioactivities and medicinal value of the fruiting body extracts of *Laetiporus sulphureus* and *Meripilus giganteus* polypore mushrooms (Agaricomycetes). Int J Med Mushrooms. 2024;26(1):17–26. 38305259 10.1615/IntJMedMushrooms.2023051297

[19] Li W. Bringing bioactive compounds into membranes: the UbiA superfamily of intramembrane aromatic prenyltransferases. Trends Biochem Sci. 2016;41(4):356–70. 26922674 10.1016/j.tibs.2016.01.007 PMC4911241

[20] Liermann JC, Thines E, Opatz T, Anke H. Drimane sesquiterpenoids from *Marasmius* sp. inhibiting the conidial germination of plant-pathogenic fungi. J Nat Prod. 2012;75(11):1983–6. 23088156 10.1021/np300337w

[21] Liu C, Minami A, Ozaki T, Wu J, Kawagishi H, Maruyama JI, et al. Efficient reconstitution of Basidiomycota diterpene erinacine gene cluster in Ascomycota host *Aspergillus oryzae* based on genomic DNA sequences. J Am Chem Soc. 2019;141(39):15519–23. 31535864 10.1021/jacs.9b08935

[22] Liu S, Tao Y, Zhang Y, Gong J, Wu Z, Wang Z, et al. Identification, characterization, and catalytic mechanism of regioselective UbiA prenyltransferases in *Morus* plants. Angew Chem Int Ed Engl. 2025;64(21):e202504190. 40080387 10.1002/anie.202504190

[23] Liu Y, Nishishita J, Liu C, Oikawa H, Minami A. Biochemistry and chemoinformatics guided classification of hirsutane sesquiterpenes isolated from mushroom. JACS Au. 2025;5(2):740–6. 40017741 10.1021/jacsau.4c00983 PMC11862936

[24] Luo P, Huang JH, Lv JM, Wang GQ, Hu D, Gao H. Biosynthesis of fungal terpenoids. Nat Prod Rep. 2024;41(5):748–83. 38265076 10.1039/d3np00052d

[25] Munakata R, Kitajima S, Nuttens A, Tatsumi K, Takemura T, Ichino T, et al. Convergent evolution of the UbiA prenyltransferase family underlies the independent acquisition of furanocoumarins in plants. New Phytol. 2020;225(5):2166–82. 31642055 10.1111/nph.16277 PMC7028039

[26] Nagamine S, Liu C, Nishishita J, Kozaki T, Sogahata K, Sato Y, et al. Ascomycete *Aspergillus oryzae* is an efficient expression host for production of Basidiomycete terpenes by using genomic DNA sequences. Appl Environ Microbiol. 2019. 10.1128/AEM.00409-19. 31101615 10.1128/AEM.00409-19 PMC6643257

[27] Nowicka B, Kruk J. Occurrence, biosynthesis and function of isoprenoid quinones. Biochim Biophys Acta. 2010;1797(9):1587–605. 20599680 10.1016/j.bbabio.2010.06.007

[28] Olennikov DN, Agafonova SV, Stolbikova AV, Rokhin AV. Melanin of *Laetiporus sulphureus* (Bull.: Fr.) Murr sterile form. Prikl Biokhim Mikrobiol. 2011;47(3):330–5. 21790034

[29] Petrović J, Glamočlija J, Stojković DS, Ćirić A, Nikolić M, Bukvički D, et al. *Laetiporus sulphureus*, edible mushroom from Serbia: investigation on volatile compounds, in vitro antimicrobial activity and in situ control of Aspergillus flavus in tomato paste. Food Chem Toxicol. 2013;59:297–302. 23811530 10.1016/j.fct.2013.06.021

[30] Petrović J, Stojković D, Reis FS, Barros L, Glamočlija J, Ćirić A, et al. Study on chemical, bioactive and food preserving properties of Laetiporus sulphureus (Bull.: Fr.) Murr. Food Funct. 2014;5(7):1441–51. 24810655 10.1039/c4fo00113c

[31] Quintero-Cabello KP, Lugo-Flores MA, Rivera-Palafox P, Silva-Espinoza BA, González-Aguilar GA, Esqueda M, et al. Antioxidant properties and industrial uses of edible Polyporales. J Fungi (Basel). 2021. 10.3390/jof7030196. 33803280 10.3390/jof7030196 PMC7998620

[32] Sampritha Devi D, Bose M, Dass RS. Chapter 11 - Antifungal compounds: With special emphasis on echinocandins, polyenes, and heterocyclic benzofurans. In: Dhara AK, Nayak AK, Chattopadhyay D, editors. Antibiotics- Therapeutic Spectrum and Limitations. London: Academic Press; 2023. p. 233–49.

[33] Sinanoglou VJ, Zoumpoulakis P, Heropoulos G, Proestos C, Ćirić A, Petrovic J, et al. Lipid and fatty acid profile of the edible fungus *Laetiporus sulphurous*. Antifungal and antibacterial properties. J Food Sci Technol. 2015;52(6):3264–72. 26028707 10.1007/s13197-014-1377-8 PMC4444868

[34] Yang E, Yao Y, Liu Y, Sun Z, Shi T, Pan Y, et al. A gatekeeper residue controls aromatic acceptor specificity of the PHB-type UbiA prenyltransferases. ACS Catal. 2023;13(20):13717–28.

[35] Yang Y, Ke N, Liu S, Li W. Methods for structural and functional analyses of intramembrane prenyltransferases in the UbiA superfamily. Methods Enzymol. 2017;584:309–47. 28065269 10.1016/bs.mie.2016.10.032 PMC5432130

[36] Yang YL, Zhou H, Du G, Feng KN, Feng T, Fu XL, et al. A monooxygenase from Boreostereum vibrans catalyzes oxidative decarboxylation in a divergent Vibralactone biosynthesis pathway. Angew Chem Int Ed Engl. 2016;55(18):5463–6. 27007916 10.1002/anie.201510928

[37] Yang YL, Zhou M, Yang L, Gressler M, Rassbach J, Wurlitzer JM, et al. A mushroom P450-monooxygenase enables regio- and stereoselective biocatalytic synthesis of epoxycyclohexenones. Angew Chem Int Ed Engl. 2023;62(49):e202313817. 37852936 10.1002/anie.202313817

[38] Yuan GY, Zhang JM, Xu QD, Zhang HR, Hu C, Zou Y. Biosynthesis of Cosmosporasides reveals the assembly line for fungal hybrid terpenoid saccharides. Angew Chem Int Ed Engl. 2023;62(41):e202308887. 37647109 10.1002/anie.202308887

