# Supplementary material for: Genome mining reveals the unique function of UbiA-type prenyltransferase in Laetiporus sulphureus
Source: Nat Prod Bioprospect. 2026 Jan 11;16(1):17. doi: 10.1007/s13659-025-00571-2 (PMC12790560; doi:10.1007/s13659-025-00571-2)
Supplement: Supplementary file 1 — Additional file 1. [file 13659_2025_571_MOESM1_ESM.docx]

**Supporting Information**

**Genome mining reveals the unique function of UbiA-type prenyltransferase in *Laetiporus sulphureus***

Yue Wang ^a^, Qian Wang ^a^, Chunlei Wang ^a^, Pengchao Wang ^a^, Ran Wang ^b^, Jing Wu ^c^, Hirokazu Kawagishi ^d^, Chengwei Liu ^a^*

^a^ State Key Laboratory of Utilization of Woody Oil Resource, College of Life Science, Northeast Forestry University, Harbin 150040, China

^b^ College of Food and Biotechnology, Changchun Polytechnic University, Changchun 130033, China

^c^ Faculty of Agriculture, Iwate University, 3-18-8 Ueda, Morioka 020-8550, Japan

^d^ Faculty of Agriculture and Research Institute of Mushroom Science, Shizuoka University, Shizuoka 422-8529, Japan

* Corresponding authors.

E-mail addresses: liuchw@nefu.edu.cn (C. Liu)

**Table of Contents**

**Supplementary Figures:**

Fig. S1 Gene structure of *LaPT (1–8)*

Fig. S2 The transmembrane prediction of LaPT (1–8)

Fig. S3 Amino acid sequence alignment of LaPT (1–8)

Fig. S4 ^1^H-NMR spectrum of **1** (CDCl_3_, 500 MHz)

Fig. S5 ^13^C-NMR spectrum of **1** (CDCl_3_, 125 MHz)

Fig. S6 HMBC spectrum of **1**

Fig. S7 HSQC spectrum of **1**

Fig. S8 NOESY spectrum of **1**

Fig. S9 ¹H-¹H COSY spectrum of **1**

**Supplementary Tables：**

Table S1 Primers used in *Aspergillus oryzae* expression vectors

Table S2 Primers for mutation analysis

Table S3 The amino acid sequence of LaPT *(1–8)*

Table S4 Information on the UbiA-type PTs used for phylogenetic analysis in Fig. 1

Table S5 Substrate Specificity of LaPTs


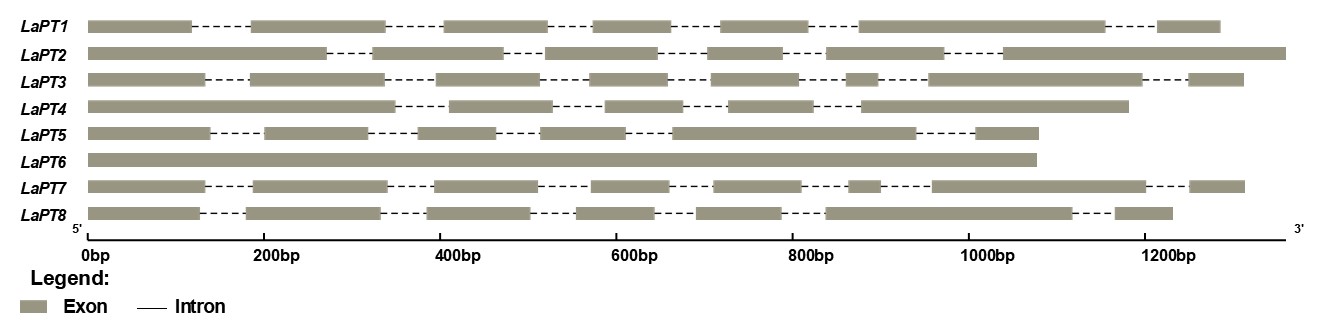


**Fig. S1** Gene structure of *LaPT(1–8)*

The squares represent exons and the lines represent introns. The genome sequence and cDNA sequence were submitted to GSDS (https://gsds.gao-lab.org/) for visual analysis.


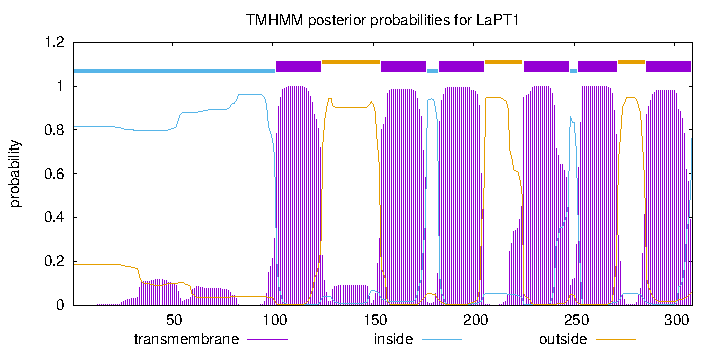

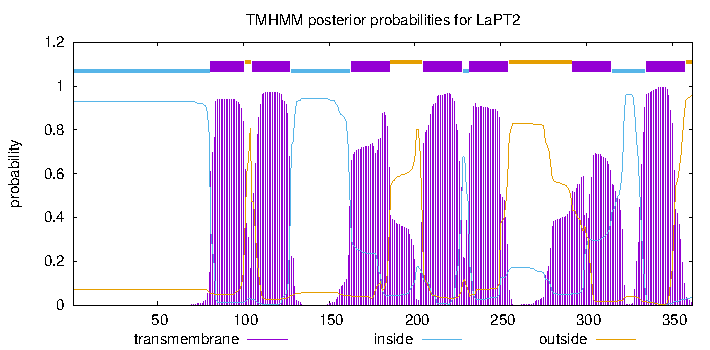


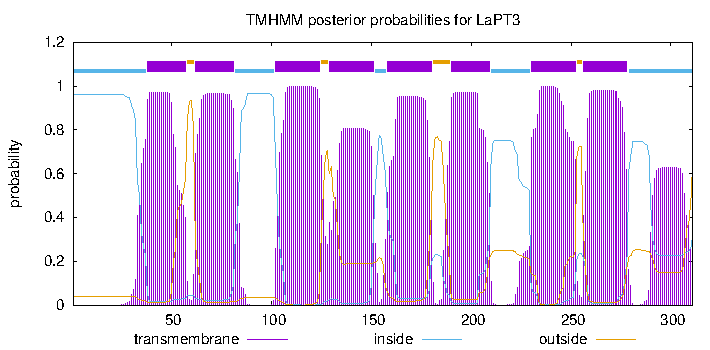

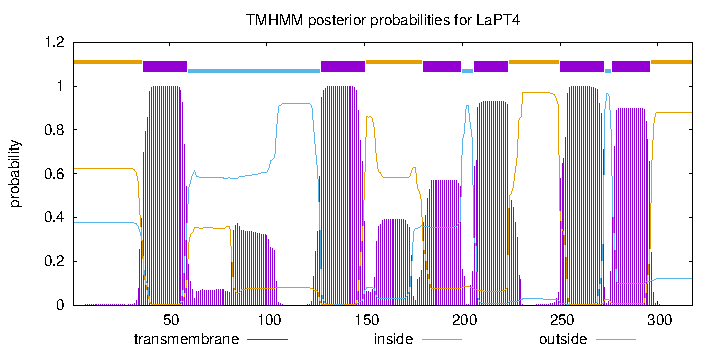


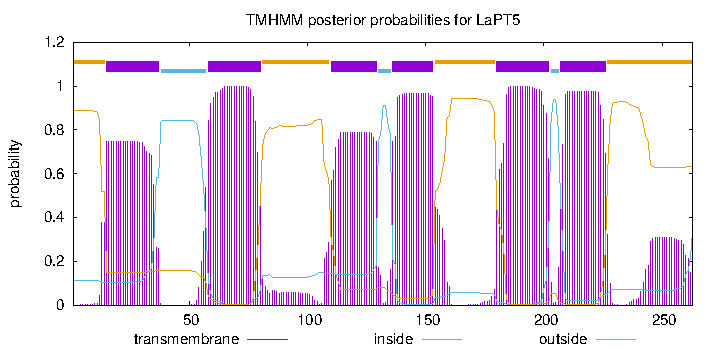

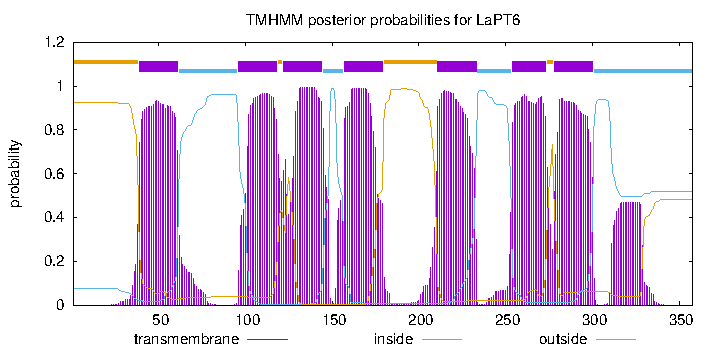


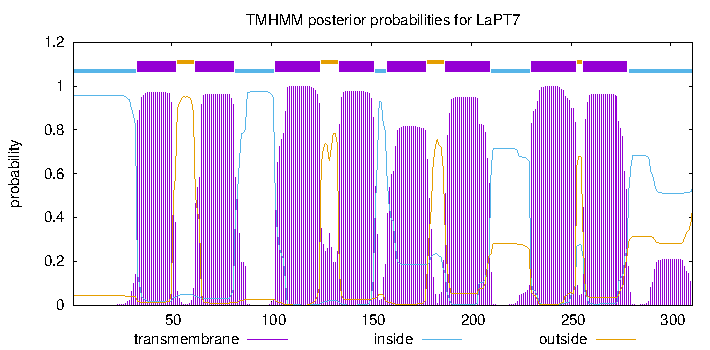

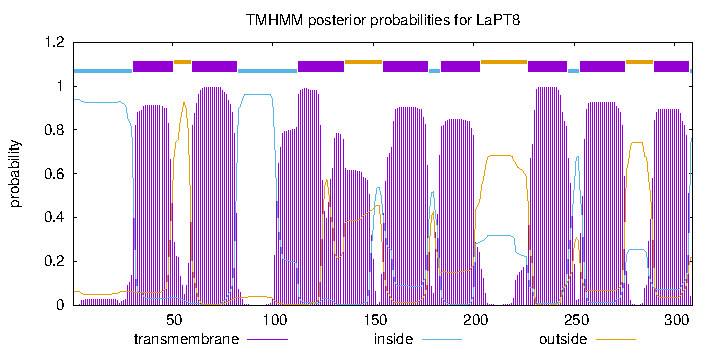


**Fig. S2** The transmembrane prediction of LaPT(1-8)

The transmembrane regions of LaPT(1-8) were predicted by the program TMHMM 2.0 (https://services.healthtech.dtu.dk/services/TMHMM-2.0/).

**
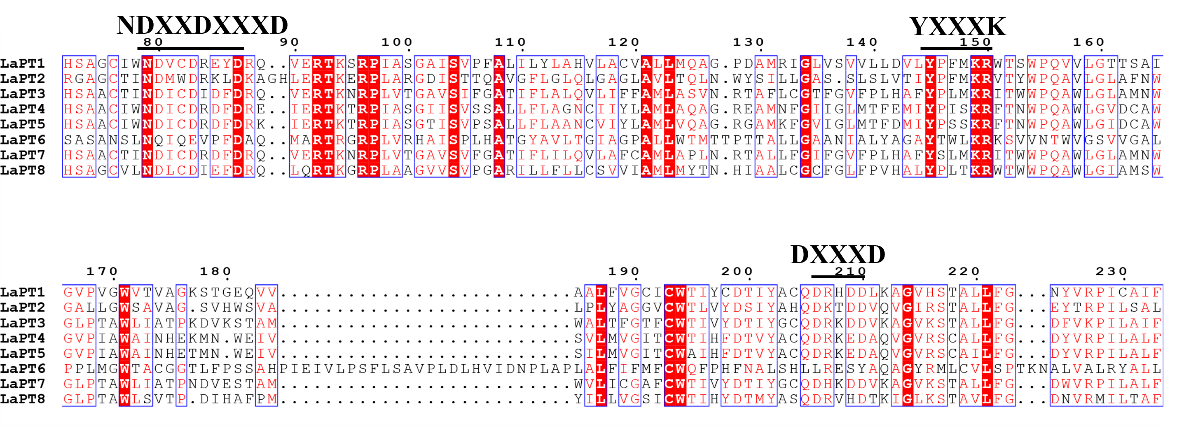
**

**Fig. S3** Amino acid sequence alignment of LaPT(1–8)

Two conserved aspartaterich motifs, NDxxDxxxD and DxxxD, and one YxxxK motif characteristic of the UbiA family are underlined.

**
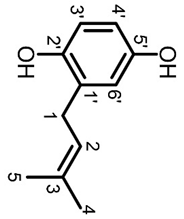
**
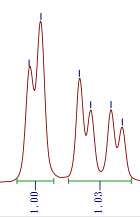

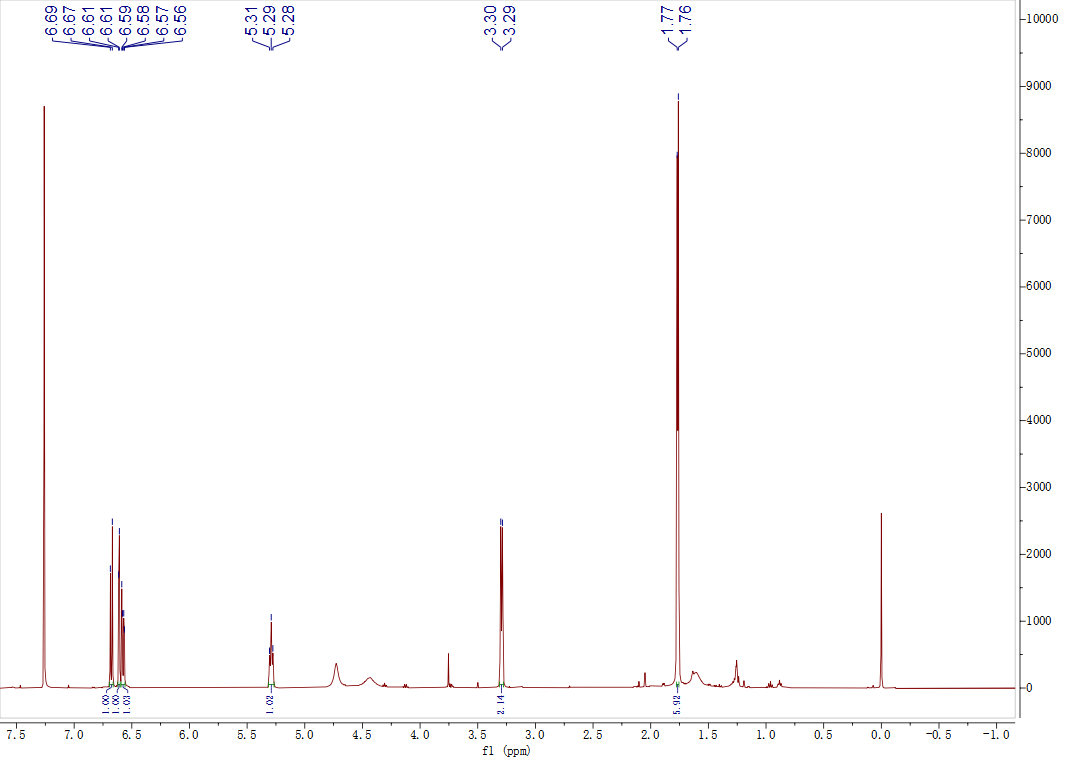


**Fig. S4** ^1^H-NMR spectrum of 1 (CDCl_3_, 500 MHz).


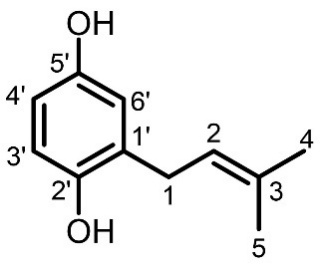

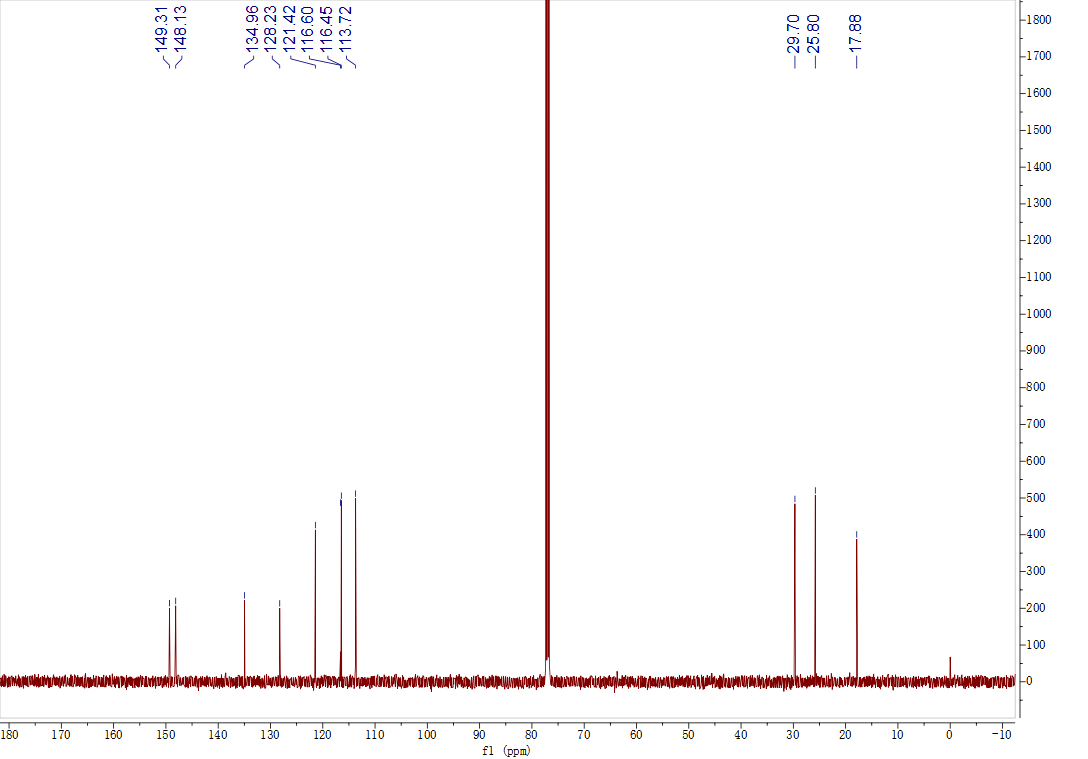


**Fig. S5** ^13^C-NMR spectrum of 1 (CDCl_3_, 125MHz).


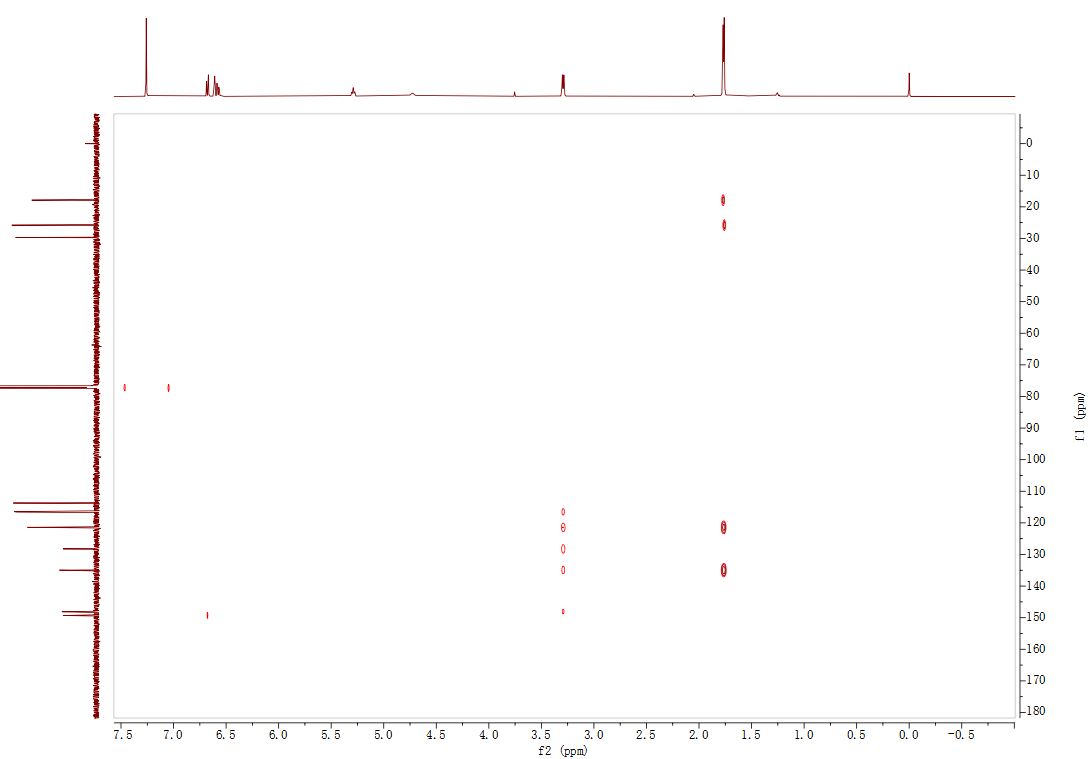


**Fig. S6** HMBC spectrum of **1**.


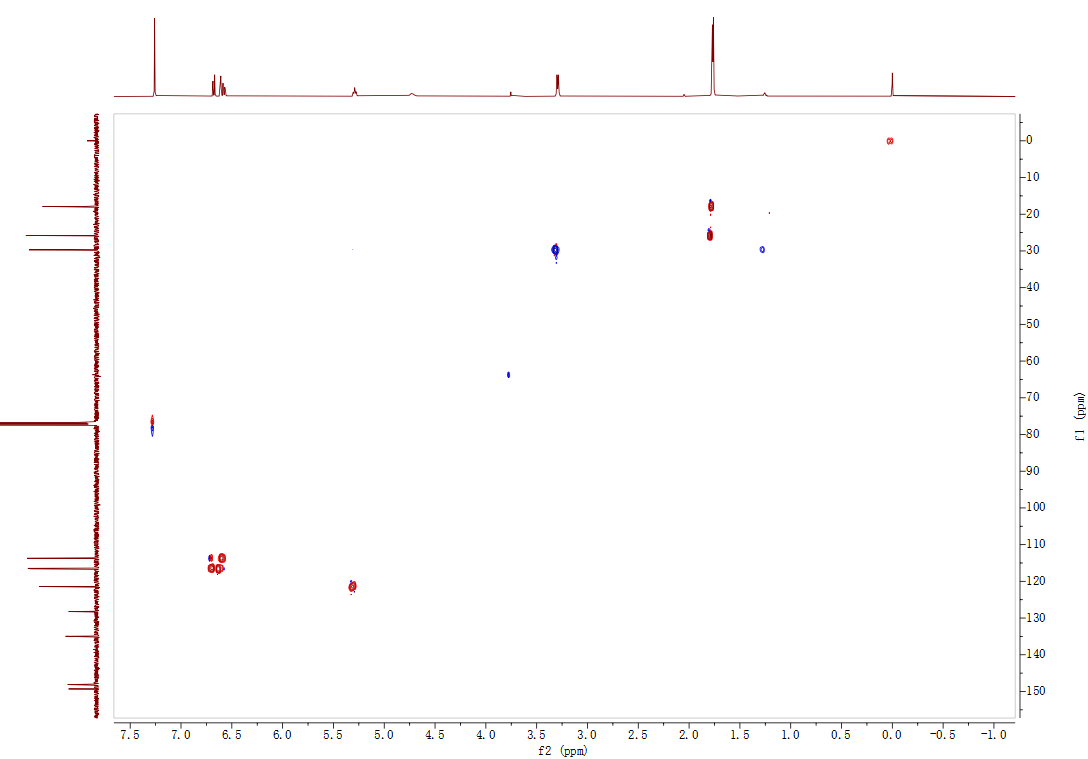


**Fig. S7** HSQC spectrum of **1**.


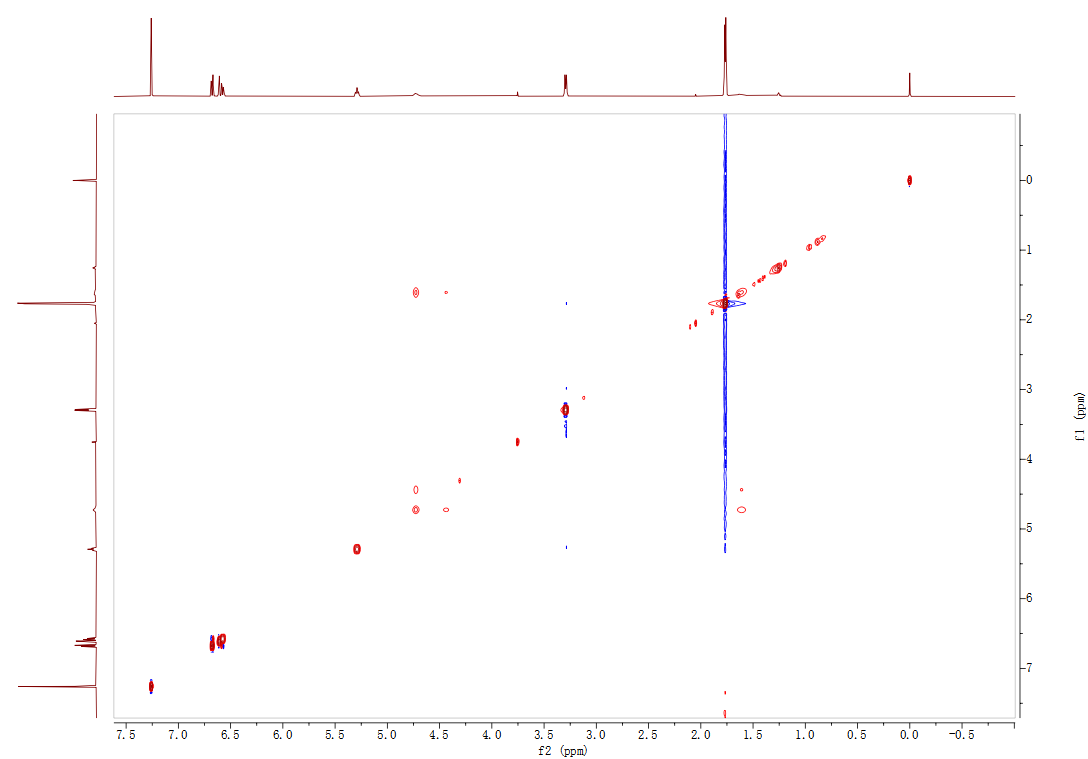


**Fig. S8** NOESY spectrum of **1**.


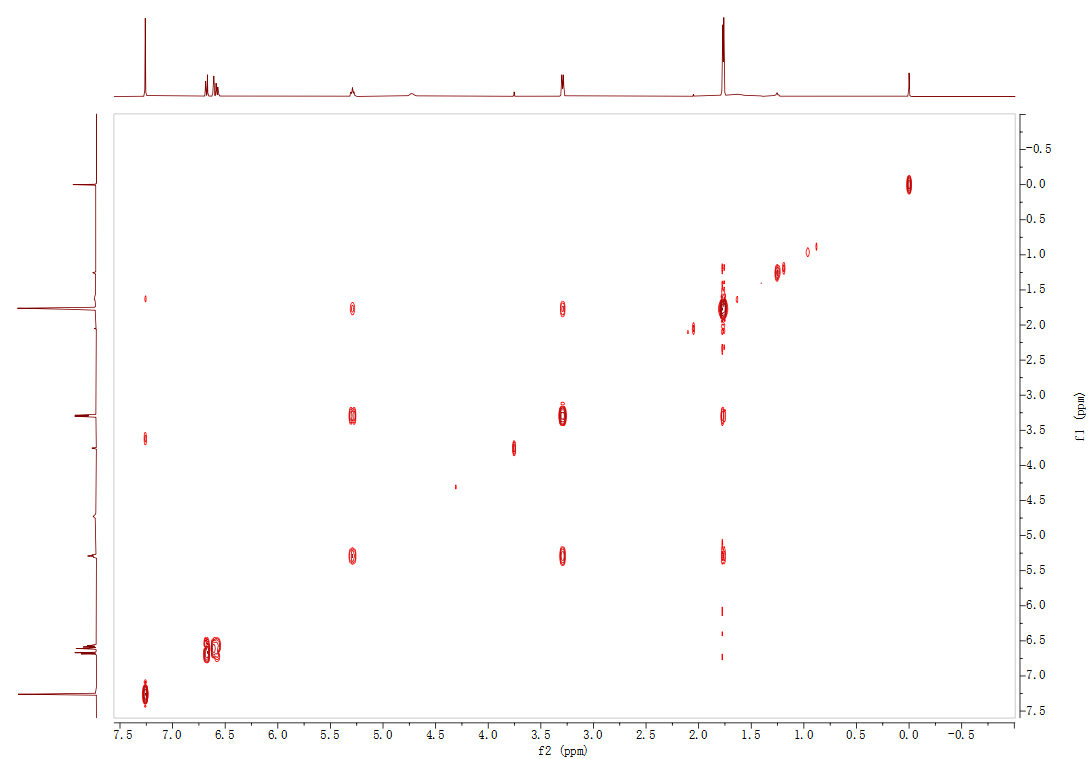


**Fig. S9** ¹H-¹H COSY spectrum of **1**.

**Table S1** Primers used in *Aspergillus oryzae* expression vectors

| Primer | Sequence（5’-3’） |
| --- | --- |
| pDP201C-*LaPT1*-F | CCGGAATTCGAGCTCGGTACCATGCACACAATGGGTACTGCAGTTG |
| pDP201C-*LaPT1*-R | ATCGGGTACGACCCGGGTACCTTAAACAATCACCCTATAGGC |
| pDP201C-*LaPT2*-F | CCGGAATTCGAGCTCGGTACCATGCACTCCTTGTTCGCCGTCCC |
| pDP201C-*LaPT2-*R | ATCGGGTACGACCCGGGTACCTCAGAAAGAGACACCGGAGATC |
| pDP201C-*LaPT3*-F | CCGGAATTCGAGCTCGGTACCATGACAGATAGTGCTGAGAAGAAG |
| pDP201C-*LaPT3*-R | ATCGGGTACGACCCGGGTACCCTAGGCGAGATAGCGCTCTGCAAG |
| pDP201C-*LaPT4*-F | CCGGAATTCGAGCTCGGTACCATGTCTAGCCAGAAGACCTCCAGC |
| pDP201C-*LaPT4*-R | ATCGGGTACGACCCGGGGTACCCTAGCGACGTGAGAAGTCTAAACC |
| pDP201C-*LaPT5*-F | CCGGAATTCGAGCTCGGTACCATGTCAGCACATGCCTCAGGGCTC |
| pDP201C-*LaPT5*-R | ATCGGGTACGACCCGGGTACCTTAAACCGCAATTTTGTAAGCATAGTCAC |
| pDP201C-*LaPT6*-F | CCGGAATTCGAGCTCGGTACCATGTCACAGTATCCCCCTTCACCC |
| pDP201C-*LaPT6*-R | ATCGGGTACGACCCGGGTACCCTACGCCTTCGTAACTGATGATG |
| pDP201C-*LaPT7*-F | CCGGAATTCGAGCTCGGTACCATGATGGACGTTGCCAAACAGGAG |
| pDP201C-*LaPT7*-R | ATCGGGTACGACCCGGGTACCCTAGGACAGGTATCGCTCTGCGATC |
| pDP201C-*LaPT8*-F | CCGGAATTCGAGCTCGGTACCATGGACCGCAAGACAGCATATTTTG |
| pDP201C-*LaPT8*-R  *LaPT3* G-A-F  *LaPT3* G-A-R  *LaPT3* N-S-F  *LaPT3* N-S-R | ATCGGGTACGACCCGGGTACCTCACCGTTTGCAAGCGTAATCGAG  ACACCATCTACGCGTGCCAGGACCGCAAG  CTGGCACGCGTAGATGGTGTCATACACGAT  AACGGACGAAGTCGAGACCCTTGGTAACTG  GGGTCTCGACTTCGTCCGTTCGACTTGACG |

**Table S2** Primers for mutation analysis

| Primer | Sequence（5’-3’） |
| --- | --- |
| *LaPT3*-77-F-A-F  *LaPT3*-77-F-A-R  *LaPT3*-80-D-A-F  *LaPT3*-80-D-A-R  *LaPT3*-81-C-A-F  *LaPT3*-81-C-A-R  *LaPT3*-84-N-A-F  *LaPT3*-84-N-A-R  *LaPT3*-88-D-A-F  *LaPT3*-88-D-A-R  *LaPT3*-92-D-A-F  *LaPT3*-92-D-A-R  *LaPT3*-97-R-A-F  *LaPT3*-97-R-A-R  *LaPT3*-101-R-A-F  *LaPT3*-101-R-A-R  *LaPT3*-150-Y-A-F  *LaPT3*-150-Y-A-R  *LaPT3*-154-K-A-F  *LaPT3*-154-K-A-R  *LaPT3*-203-Y-A-F  *LaPT3*-203-Y-A-R  *LaPT3*-204-D-A-F  *LaPT3*-204-D-A-R  *LaPT3*-207-Y-A-F  *LaPT3*-207-Y-A-R  *LaPT3*-211-D-A-F  *LaPT3*-211-D-A-R  *LaPT3*-215-D-A-F  *LaPT3*-215-D-A-R  *LaPT3* G-A-F  *LaPT3* G-A-R  *LaPT3* N-S-F  *LaPT3* N-S-R | ACACTGCTGGCCAGTGCGGCCTGTA  CCGCACTGCCCAGCAGTGTGCTGCC  ACAGTGCGCACTGTACCATCAACGAC  ATGGTACAGCACGCACTGTGCAGCAG  AGTGCGGCCGCCACCATCAACGACATC  TTGATGGTGGCGGCCGCACTGTGCAGC  CCTGTACCATCGCCGACATCTGCGATATA  ATGTCGGCGATGGTACAGGCCGCACTGTGC  ACGACATCTGCGCCATAGACTTTGACCGT  AAGTCTATGGCGCAGATGTCGTTGATGGTA  TATAGACTTTGCCCGTCAAGTCGAACGGA  ACTTGACGGGCAAAGTCTATATCGCAGAT  TCAAGTCGAACCGACGAAGAATAGACCCT  TATTCTTCGTCGGTTCGACTTGACGGTCA  CGAAGAATGCACCCTTGGTAACTGG  CCAAGGGTGCATTCTTCGTCCGTTCGA  ACGCGTTCGCCCCACTTATGAAACGC  ATAAGTGGGGCGAACGCGTGCAGTGG  TCCACTTATGGCACGCATCACCTGGTG  ATGCGTGCCATAAGTGGATAGAACGCG  ACTATCGTGGCCGACACCATCTACGGC  ATGGTGTCGGCCACGATAGTCCAGCAG  CGTGTATGCCACCATCTACGGCTGC  AGATGGTGGCATACACGATAGTCCA  TGACACCATCGCCGGCTGCCAGGACCGCA  CTGGCAGCCGGCGATGGTGTCATACACGA  ACGGCTGCCAGGCCCGCAAGGACGATGT  TCCTTGCGGGCCTGGCAGCCGTAGATGGT  CAAGGACGCCGTCAAAGCGGGCGTGA  GCTTTGACGGCGTCCTTGCGGTCCTG  ACACCATCTACGCGTGCCAGGACCGCAAG  CTGGCACGCGTAGATGGTGTCATACACGAT  AACGGACGAAGTCGAGACCCTTGGTAACTG  GGGTCTCGACTTCGTCCGTTCGACTTGACG |

**Table S3** The amino acid sequence of *LaPT(1–8)*

| Gene | amino acid sequence |
| --- | --- |
| *LaPT1* | MHTMGTAVDNAHPPLWRSLLQLTRLHLFPLGPDLAFWPSAWGLTLSVCATGLPPKQLANLSFWFFVGGTLRHSAGCIWNDVCDREYDRQVERTKSRPIASGAISVPFALILYLAHVLACVALLMQAGPDAMRIGLVSVVLLDVLYPFMKRWTSWPQVVLGTTSAIGVPVGWVTVAGKSTGEQVVAALFVGCICWTIYCDTIYACQDRHDDLKAGVHSTALLFGNYVRPICAIFAAIFVACMATAGALNGQGPLFFIITVLGTAVTLAWQMATVDLDNGADCMKAFMVNAYLSYGMWAGMLLDYAYRVIV |
| *LaPT2* | MHSLFAVPRRTYILSGLNAHRRLSCLQNRNLIPLVRLASTKVTVRQNATLSSPSTAAKSWVEHLPAKIRPYLYLTRIDKPIGTLLLFYPCTWSITMASYALQTPWTTPLTYISLFGIGALVMRGAGCTINDMWDRKLDKAGHLERTKERPLARGDISTTQAVGFLGLQLGAGLAVLTQLNWYSILLGASSLSLVTIYPFMKRVTYWPQAVLGLAFNWGALLGWSAVAGSVHWSVALPLYAGGVCWTLVYDSIYAHQDKTDDVQVGIRSTALLFGEYTRPILSALSVSSLSLITYAGYLNAQGPLFYTGTGLAALQLARVLVRTDFDNRPSCWAGFVGCGWSGFWIWMGALADYGFMISGVSF |
| *LaPT3* | MTDSAEKKMSTNSAGPSPSWWRPYWELARMHKFPAGSILVFWPCVWGYLLSPGSAGLSPRELAVHIFALLVGSTLLHSAACTINDICDIDFDRQVERTKNRPLVTGAVSIFGATIFLALQVLIFFAMLASVNRTAFLCGTFGVFPLHAFYPLMKRITWWPQAWLGLAMNWGLPTAWLIATPKDVKSTAMWALTFGTFCWTIVYDTIYGCQDRKDDVKAGVKSTALLFGDFVKPILAIFAAIFVASLGYAGVVTGRSPIYFVLAVGGCAAHLAWQLLTLRAEDPKDCWRKFNANGYLGYIVAGGMLAERYLA |
| *LaPT4* | MSSQKTSSNIIPTATHRSAPPLWQSYLKLARMHTWPAGTLLFFLPCSAATSFLLLVLVANNCRLAAWALAMSAYTSGLPPKHLFIQGLGCLLMCTVRHSAACIWNDICDRDFDREIERTKTRPIASGIISVSSALLFLAGNCIIYLAMLAQAGREAMNFGIIGLMTFEMIYPISKRFTNWPQAWLGVDCAWGVPIAWAINHEKMNWEIVSVLMVGITCWTIHFDTVYACQDRKEDAQVGVRSCALLFGDYVRPILALFSTAFIASLAYAGYLNRQGSLYYIIAVLGSASHLVWQFASSDDWERDGGRIWKVGLDFSRR |
| *LaPT5* | MSAHASGLPPEHLLIQGLGCLLMCTVRHSAACIWNDICDRDFDRKIERTKTRPIASGTISVPSALLFLAANCVIYLAMLVQAGRGAMKFGVIGLMTFDMIYPSSKRFTNWPQAWLGIDCAWGVPIAWAINHETMNWEIVSILMVGITCWAIHFDTVYACQDRKEDAQVGVRSCAILFGDYVRPILALFSTAFIASLAYAGYLNRQGPLYYIIAVLGSALHLVWQFASSDDWEKDGGRIFKSNGDLGYIVLAGMLCDYAYKIAV |
| *LaPT6* | MSQYPPSPAVYPYKQTEAMTSRQLLKVYWQLSKPHLSMFVILAAMSGVALSPLPTTVPVLLSTAIGTALCSASANSLNQIQEVPFDAQMARTRGRPLVRHAISPLHATGYAVLTGIAGPALLWTMTTPTTALLGAANIALYAGAYTWLKRKSVVNTWVGSVVGALPPLMGWTACGGTLFPSSAHPIEIVLPSFLSAVPLDLHVIDNPLAPLALFIFMFCWQFPHFNALSHLLRESYAQAGYRMLCVLSPTKNALVALRYALLPIPLCSILIPLSGLTSWTFAIVSLVPNAICAEAAWRFWRNGRDKDARRLFQHSLWYLPVMLGLMMICKRGLDWGSWLGTQEDGEEEARESSSVTKA |
| *LaPT7* | MMDVAKQEATVQLKGSAPLQWQAYWELARMHKFPAGSILVFWPCVWGYLLSSGSDKLTSRELASHILAFLIGSTLLHSAACTINDICDRDFDRQVERTKNRPLVTGAVSVFGATIFLILQVLAFCAMLAPLNRTALLFGIFGVFPLHAFYSLMKRITWWPQAWLGLAMNWGLPTAWLIATPNDVESTAMWVLICGAFCWTIVYDTIYGCQDHKDDVKAGVKSTALLFGDWVRPILALFAAVFVASLTYASIVTGQSWFYFVLTVGGCALHLVWQLVTLEVDNPEDCWRKFKANGSLGYIVAGGMIAERYLS |
| *LaPT8* | MDRKTAYFASLPFLAQFPWRGYYELTRLHKSLLGNALVFWPCAWGLTMSSYRVNAPAVPFMIQTLVFAVGCIFLHSAGCVLNDLCDIEFDRQLQRTKGRPLAAGVVSVPGARILLFLLCSVVIAMLMYTNHIAALCGCFGLFPVHALYPLTKRWTWWPQAWLGIAMSWGLPTAWLSVTPDIHAFPMYILLVGSICWTIHYDTMYASQDRVHDTKIGLKSTAVLFGDNVRMILTAFCMVFLVSLVMAGHYNNQSMLYYIFSCGGAAAHLCWQLLTWQTSEMKDSDAKYQSNGMVGLIIWMGMILDYACKR |

**Table S4** Information on the UbiA-type PTs used for phylogenetic analysis in Fig. 1

| **Species** | **Enzyme** | **Genebank** | **References** |
| --- | --- | --- | --- |
| Aspergillus novofumigatus | NvfB | A0A2I1BT09.1 | [1] |
| Acremonium egyptiacum | AscA | A0A455R413.1 | [2] |
| Nectria sp. | NtnF | A0A455LM21.1 | [3] |
| Arthrinium sp. | AtnF | A0A455LRX2.1 |  |
| Talaromyces verruculosus | CdmH | A0A3G9GNJ4.1 | [4] |
| Colletotrichum higginsianum | DpchC | A0A1B7YCK2.1 | [5] |
| Fusarium graminearum PH-1 | DpfgC | I1RL16.1 | [6] |
| Apiospora sacchari | DpasC | P9WEX2.1 | [7] |
| Macrophomina phaseolina | DpmpC | P9WEW7.1 | [8] |
| Penicillium canescens | OlcH | P9WEQ7.1 | [9] |
| Aspergillus terreus | Trt2 | Q0C8A6.1 | [10] |
| Aspergillus nidulans | AusN | Q5AR21.1 | [11] |
|  | PkfE | Q5B8A2.1 |  |
| Aspergillus fumigatus | Pyr6 | Q4WLD0.2 | [12] |
| Penicillium brevicompactum | PgMpaA | XP_056812872.1 | [13] |
| Penicillium roqueforti | AdrG | A0A1Y0BRF7.1 | [14] |
| Aspergillus stellatus | AndD | A0A097ZPE3.1 | [15] |
| Aspergillus niger | YanG | G3Y418.1 | [16] |
| Fusarium tricinctum | CosA | KAH7245299.1 | [17] |
| Coleophoma cylindrospora | ColA | RDW88428.1 | [18] |
| *Panus rudis* | PanE | ON185534 | [19] |
| *Hohenbuehelia grisea* | *HGRIS* | KAL0957959.2 | [20] |
| *Boreostereum vibrans* | VibP1 | ON653009 | [21] |
|  | VibP2 | ON653010 |  |
| *Stereum hirsutum* | *Sh*PT1 | XP_007303462.1 |  |
|  | *ShPT2* | XP_007299647.1 |  |
| *Clitocybe clavipes* | *ClaS* | P9WEI6.1 | [22] |
| *Gautieria morchelliformis* | *GmUbiA* | KAF8510743.1 |  |
| *Auriscalpium vulgare* | *AvUbiA* | KAI0040628.1 |  |

**Table S5** Substrate Specificity of LaPTs

| 底物 | LaPT1 | LaPT2 | LaPT3 | LaPT4 | LaPT5 | LaPT6 | LaPT7 | LaPT8 |
| --- | --- | --- | --- | --- | --- | --- | --- | --- |
| Hydroquinone | **_-** | **_-** | **+** | **_-** | **_-** | **_-** | **_-** | **_-** |
| 4-Hydroxybenzoic acid | **_-** | **_-** | **_-** | **_-** | **_-** | **_-** | **_-** | **_-** |
| p-hydroxybenzyl alcohol | **_-** | **_-** | **_-** | **_-** | **_-** | **_-** | **_-** | **_-** |
| Olivanic acid | **_-** | **_-** | **_-** | **_-** | **_-** | **_-** | **_-** | **_-** |
| o-Orsellinaldehyde | **_-** | **_-** | **_-** | **_-** | **_-** | **_-** | **_-** | **_-** |
| 4-Hydroxybenzaldehyde | **_-** | **_-** | **_-** | **_-** | **_-** | **_-** | **_-** | **_-** |
| Phloroglucinol | **_-** | **_-** | **_-** | **_-** | **_-** | **_-** | **_-** | **_-** |
| 1-Hydroxynaphthalen | **_-** | **_-** | **_-** | **_-** | **_-** | **_-** | **_-** | **_-** |

References

1. Matsuda, Y.; Bai, T.; Phippen, C. B. W.; Nødvig, C. S.; Kjærbølling, I.; Vesth, T. C.; Andersen, M. R.; Mortensen, U. H.; Gotfredsen, C. H.; Abe, I.; et al. Novofumigatonin biosynthesis involves a non-heme iron-dependent endoperoxide isomerase for orthoester formation. Nat Commun 2018, 9 (1), 2587.

2. Araki, Y.; Awakawa, T.; Matsuzaki, M.; Cho, R.; Matsuda, Y.; Hoshino, S.; Shinohara, Y.; Yamamoto, M.; Kido, Y.; Inaoka, D. K.; et al. Complete biosynthetic pathways of ascofuranone and ascochlorin in Acremonium egyptiacum. Proc Natl Acad Sci U S A 2019, 116 (17), 8269-8274.

3. Zhang, X.; Wang, T. T.; Xu, Q. L.; Xiong, Y.; Zhang, L.; Han, H.; Xu, K.; Guo, W. J.; Xu, Q.; Tan, R. X.; et al. Genome Mining and Comparative Biosynthesis of Meroterpenoids from Two Phylogenetically Distinct Fungi. Angew Chem Int Ed Engl 2018, 57 (27), 8184-8188.

4. Bai, T.; Quan, Z.; Zhai, R.; Awakawa, T.; Matsuda, Y.; Abe, I. Elucidation and Heterologous Reconstitution of Chrodrimanin B Biosynthesis. Org Lett 2018, 20 (23), 7504-7508.

5. O'Connell, R. J.; Thon, M. R.; Hacquard, S.; Amyotte, S. G.; Kleemann, J.; Torres, M. F.; Damm, U.; Buiate, E. A.; Epstein, L.; Alkan, N.; et al. Lifestyle transitions in plant pathogenic Colletotrichum fungi deciphered by genome and transcriptome analyses. Nat Genet 2012, 44 (9), 1060-1065.

6. Cuomo, C. A.; Güldener, U.; Xu, J. R.; Trail, F.; Turgeon, B. G.; Di Pietro, A.; Walton, J. D.; Ma, L. J.; Baker, S. E.; Rep, M.; et al. The Fusarium graminearum genome reveals a link between localized polymorphism and pathogen specialization. Science 2007, 317 (5843), 1400-1402.

7. Tsukada, K.; Shinki, S.; Kaneko, A.; Murakami, K.; Irie, K.; Murai, M.; Miyoshi, H.; Dan, S.; Kawaji, K.; Hayashi, H.; et al. Synthetic biology based construction of biological activity-related library of fungal decalin-containing diterpenoid pyrones. Nat Commun 2020, 11 (1), 1830.

8. Islam, M. S.; Haque, M. S.; Islam, M. M.; Emdad, E. M.; Halim, A.; Hossen, Q. M.; Hossain, M. Z.; Ahmed, B.; Rahim, S.; Rahman, M. S.; et al. Tools to kill: genome of one of the most destructive plant pathogenic fungi Macrophomina phaseolina. BMC Genomics 2012, 13, 493.

9. Yaegashi, J.; Romsdahl, J.; Chiang, Y. M.; Wang, C. C. C. Genome mining and molecular characterization of the biosynthetic gene cluster of a diterpenic meroterpenoid, 15-deoxyoxalicine B, in Penicillium canescens. Chem Sci 2015, 6 (11), 6537-6544.

10. Itoh, T.; Tokunaga, K.; Radhakrishnan, E. K.; Fujii, I.; Abe, I.; Ebizuka, Y.; Kushiro, T. Identification of a key prenyltransferase involved in biosynthesis of the most abundant fungal meroterpenoids derived from 3,5-dimethylorsellinic acid. Chembiochem 2012, 13 (8), 1132-1135.

11. Galagan, J. E.; Calvo, S. E.; Cuomo, C.; Ma, L. J.; Wortman, J. R.; Batzoglou, S.; Lee, S. I.; Baştürkmen, M.; Spevak, C. C.; Clutterbuck, J.; et al. Sequencing of Aspergillus nidulans and comparative analysis with A. fumigatus and A. oryzae. Nature 2005, 438 (7071), 1105-1115.

12. Nierman, W. C.; Pain, A.; Anderson, M. J.; Wortman, J. R.; Kim, H. S.; Arroyo, J.; Berriman, M.; Abe, K.; Archer, D. B.; Bermejo, C.; et al. Genomic sequence of the pathogenic and allergenic filamentous fungus Aspergillus fumigatus. Nature 2005, 438 (7071), 1151-1156.

13. Petersen, C.; Sørensen, T.; Nielsen, M. R.; Sondergaard, T. E.; Sørensen, J. L.; Fitzpatrick, D. A.; Frisvad, J. C.; Nielsen, K. L. Comparative genomic study of the Penicillium genus elucidates a diverse pangenome and 15 lateral gene transfer events. IMA Fungus 2023, 14 (1), 3.

14. Rojas-Aedo, J. F.; Gil-Durán, C.; Del-Cid, A.; Valdés, N.; Álamos, P.; Vaca, I.; García-Rico, R. O.; Levicán, G.; Tello, M.; Chávez, R. The Biosynthetic Gene Cluster for Andrastin A in Penicillium roqueforti. Front Microbiol 2017, 8, 813.

15. Matsuda, Y.; Wakimoto, T.; Mori, T.; Awakawa, T.; Abe, I. Complete biosynthetic pathway of anditomin: nature's sophisticated synthetic route to a complex fungal meroterpenoid. J Am Chem Soc 2014, 136 (43), 15326-15336.

16. Andersen, M. R.; Salazar, M. P.; Schaap, P. J.; van de Vondervoort, P. J.; Culley, D.; Thykaer, J.; Frisvad, J. C.; Nielsen, K. F.; Albang, R.; Albermann, K.; et al. Comparative genomics of citric-acid-producing Aspergillus niger ATCC 1015 versus enzyme-producing CBS 513.88. Genome Res 2011, 21 (6), 885-897.

17. Mesny, F.; Miyauchi, S.; Thiergart, T.; Pickel, B.; Atanasova, L.; Karlsson, M.; Hüttel, B.; Barry, K. W.; Haridas, S.; Chen, C.; et al. Genetic determinants of endophytism in the Arabidopsis root mycobiome. Nat Commun 2021, 12 (1), 7227.

18. Wingfield, B. D.; Bills, G. F.; Dong, Y.; Huang, W.; Nel, W. J.; Swalarsk-Parry, B. S.; Vaghefi, N.; Wilken, P. M.; An, Z.; de Beer, Z. W.; et al. IMA Genome-F 9: Draft genome sequence of Annulohypoxylon stygium, Aspergillus mulundensis, Berkeleyomyces basicola (syn. Thielaviopsis basicola), Ceratocystis smalleyi, two Cercospora beticola strains, Coleophoma cylindrospora, Fusarium fracticaudum, Phialophora cf. hyalina, and Morchella septimelata. IMA Fungus 2018, 9 (1), 199-223.

19. Yang, Y. L.; Zhou, M.; Yang, L.; Gressler, M.; Rassbach, J.; Wurlitzer, J. M.; Zeng, Y.; Gao, K.; Hoffmeister, D. A Mushroom P450-Monooxygenase Enables Regio- and Stereoselective Biocatalytic Synthesis of Epoxycyclohexenones. Angew Chem Int Ed Engl 2023, 62 (49), e202313817.

20. Weaver, J. A.; Alkhder, D.; Prasongpholchai, P.; Tadesse, M. D.; de Los Santos, E. L.; Song, L.; Corre, C.; Alberti, F. Early Steps of the Biosynthesis of the Anticancer Antibiotic Pleurotin. ACS Chem Biol 2024, 19 (11), 2284-2290.

21. Feng, K. N.; Zhang, Y.; Zhang, M.; Yang, Y. L.; Liu, J. K.; Pan, L.; Zeng, Y. A flavin-monooxygenase catalyzing oxepinone formation and the complete biosynthesis of vibralactone. Nat Commun 2023, 14 (1), 3436.

22. Yang, E.; Yao, Y.; Liu, Y.; Sun, Z.; Shi, T.; Pan, Y.; Gao, S.-S.; Xu, X.; Ma, G.; Liu, G. A Gatekeeper Residue Controls Aromatic Acceptor Specificity of the PHB-Type UbiA Prenyltransferases. ACS Catalysis 2023, 13 (20), 13717-13728.
